# Assignment of unimodal probability distribution models for quantitative morphological phenotyping

**DOI:** 10.1186/s12915-022-01283-6

**Published:** 2022-03-31

**Authors:** Farzan Ghanegolmohammadi, Shinsuke Ohnuki, Yoshikazu Ohya

**Affiliations:** 1 Department of Integrated Biosciences, Graduate School of Frontier Sciences, The University of Tokyo, Bldg. FSB-101, 5-1-5 Kashiwanoha, Kashiwa, Chiba Prefecture 277-8562 Japan; 2 Department of Biological Engineering, Massachusetts Institute of Technology, Cambridge, MA 02139 USA; 3 Collaborative Research Institute for Innovative Microbiology, The University of Tokyo, 1-1-1 Yayoi, Bunkyo-ku, Tokyo, 113-8657 Japan

**Keywords:** CalMorph, Morphological profile, Phenome, *Saccharomyces cerevisiae*

## Abstract

**Background:**

Cell morphology is a complex and integrative readout, and therefore, an attractive measurement for assessing the effects of genetic and chemical perturbations to cells. Microscopic images provide rich information on cell morphology; therefore, subjective morphological features are frequently extracted from digital images. However, measured datasets are fundamentally noisy; thus, estimation of the true values is an ultimate goal in quantitative morphological phenotyping. Ideal image analyses require precision, such as proper probability distribution analyses to detect subtle morphological changes, recall to minimize artifacts due to experimental error, and reproducibility to confirm the results.

**Results:**

Here, we present UNIMO (UNImodal MOrphological data), a reliable pipeline for precise detection of subtle morphological changes by assigning unimodal probability distributions to morphological features of the budding yeast cells. By defining the data type, followed by validation using the model selection method, examination of 33 probability distributions revealed nine best-fitting probability distributions. The modality of the distribution was then clarified for each morphological feature using a probabilistic mixture model. Using a reliable and detailed set of experimental log data of wild-type morphological replicates, we considered the effects of confounding factors. As a result, most of the yeast morphological parameters exhibited unimodal distributions that can be used as basic tools for powerful downstream parametric analyses. The power of the proposed pipeline was confirmed by reanalyzing morphological changes in non-essential yeast mutants and detecting 1284 more mutants with morphological defects compared with a conventional approach (Box–Cox transformation). Furthermore, the combined use of canonical correlation analysis permitted global views on the cellular network as well as new insights into possible gene functions.

**Conclusions:**

Based on statistical principles, we showed that UNIMO offers better predictions of the true values of morphological measurements. We also demonstrated how these concepts can provide biologically important information. This study draws attention to the necessity of employing a proper approach to do more with less.

**Supplementary Information:**

The online version contains supplementary material available at 10.1186/s12915-022-01283-6.

## Background

Morphology is a basic phenotypic characteristic that can be affected by genetic and environmental perturbations. Consequently, living organisms have their own morphologies that evolve through natural selection [1, 2]. In addition to the morphology of individuals, morphology can also be defined at the cellular level. Cell morphology is frequently related to cellular function. While cell morphology reflects the behaviors and intercellular communication of cells in a multicellular organism, it is affected by the genotype and genetic networks in unicellular organisms [3]. Therefore, morphological phenotyping of unicellular organisms has been carried out to achieve a global understanding of the cell system as well as to answer specific questions in cell biology [4, 5].

**Fig. 1:**
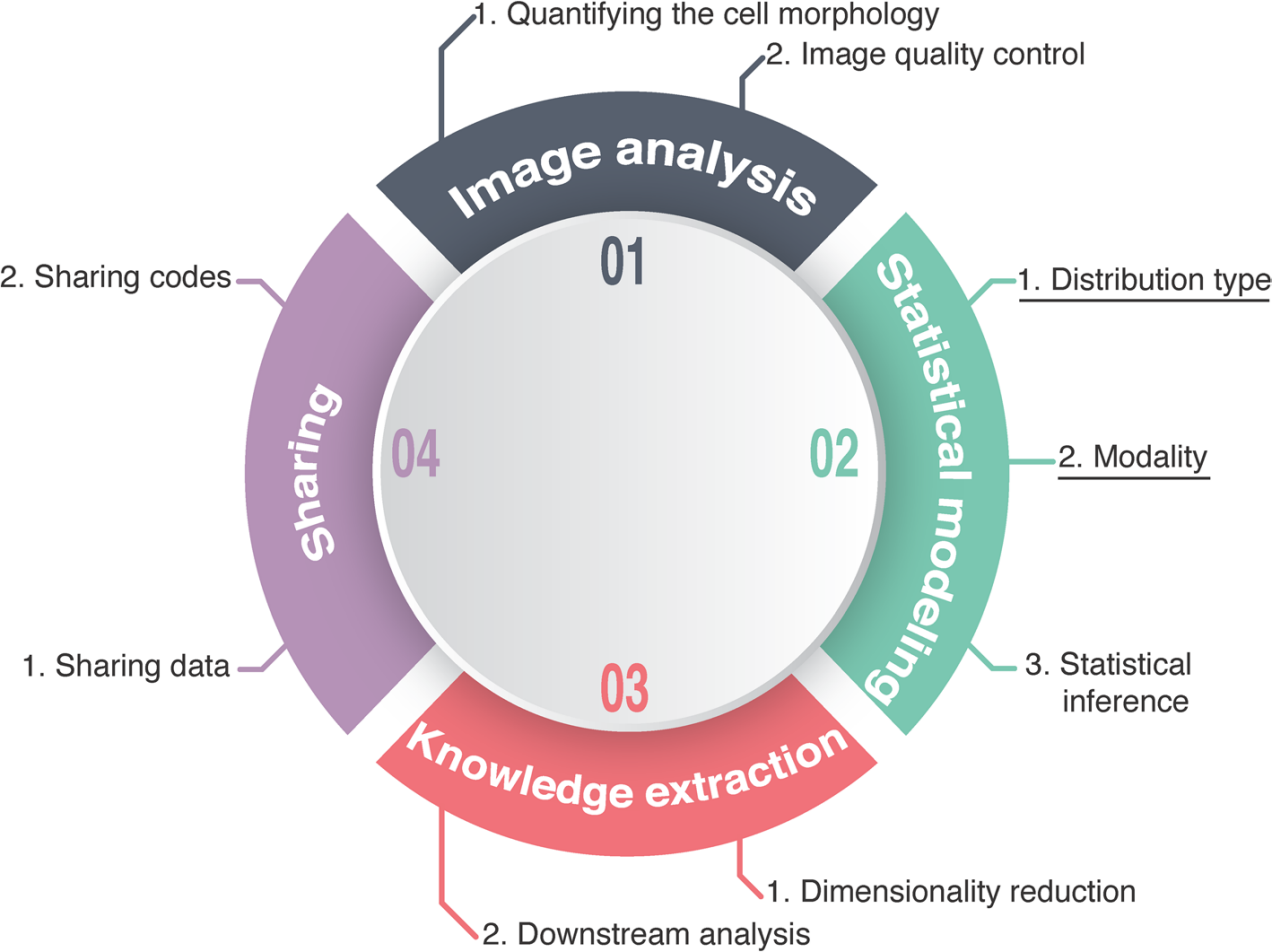
QMP pipeline. Representative workflow of the main steps for QMP. Each step represents a wide range of analytical methods and various approaches that might vary from study to study [16]. The underlined terms represent the main focus of this paper

Quantitative morphological phenotyping (QMP; Fig. 1) of cells is performed using a well-designed procedure [6–8]. Following precise sample preparation [9], the QMP pipeline begins with image analysis (Fig. 1-01). Morphological information can be obtained by processing digital images of cells. Morphological features are extracted using various image analysis tools [10, 11]. For example, CellProfiler is used for analyzing images of the tissues and cells of humans, fruit flies, worms, and yeast [12], while CalMorph is used for analyzing images of *Saccharomyces cerevisiae* cells [9, 13–15]. Increased reproducibility and reduced artifacts are often achieved using image quality control systems associated with the image analysis tools [16]. After statistical modeling by transforming and normalizing data (Fig. 1-02), the morphological data are interpreted as biologically meaningful insights (Fig. 1-03) and shared by the community, finally inspiring collaborative advancements (Fig. 1-04) [17]. Although many data analysis techniques have been developed, more precise and accurate morphological analytical methods are still desired for QMP.

**Fig. 2:**
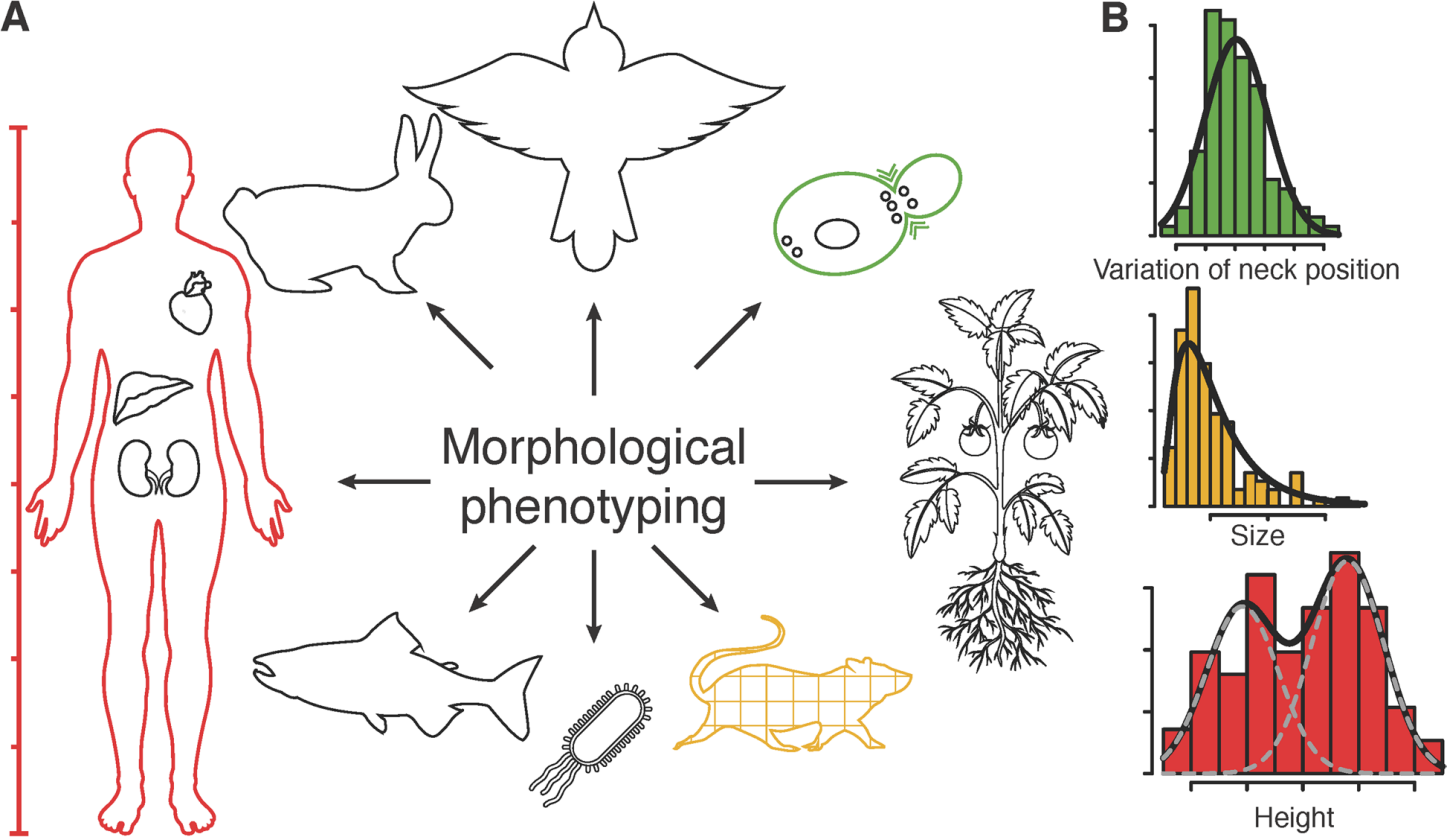
Possible morphological features used in QMP. **A** Morphological phenotyping includes a wide range of research topics and, in each field, includes various morphometric, densitometric, and structural/spatial features. **B** Examples of different types of distributions (bell-shaped, skewed, and multimodal) that can be found in morphological measurements are shown. For accurate biological inference, these characteristics must be properly addressed. This figure was designed using resources from www.freepik.com

Because biological measurements are fundamentally noisy, efforts have been made to estimate true values in quantitative biology [7, 18, 19]. The true value of a measurement can be statistically estimated with an appropriate probability distribution, which is defined as the mapping between a measurable space and the unitary interval. Commonly used probability distributions for biological data are unimodal: Gaussian, reverse Gumbel, gamma, beta, etc; distributions for continuous variables, as well as binomial, beta-binomial, Poisson, hypergeometric, etc.; and distributions for discrete variables [20]. However, it is uncertain whether real biological data, such as microscope images, follow a simple probability distribution. If a probability distribution behaves unimodally in the morphology space, a precise and accurate estimation of the true value of a morphological measure can be obtained (Fig. 2).

To precisely estimate the true values of morphological data, we attempted to assign the best-fit unimodal probability distribution model for each morphological measure. After appropriate probability distribution models were logically assigned to each of the 501 subjective morphological features defined in *S. cerevisiae* [9], we validated them using the Akaike information criterion (AIC) [21]. We then examined the modality of the distribution of the morphological features. We used existing approaches and R packages to integrate these sequential steps into a single logical pipeline (UNImodal Morphological [UNIMO)] data). Through the successful assignment of unimodal probability distribution models, we can run a powerful parametric approach, providing new biological information that can be masked in commonly used methods. Our research highlights the importance of employing proper analytical tools in phenome studies.

## Results

### Probability distribution in morphological features

**Fig. 3:**
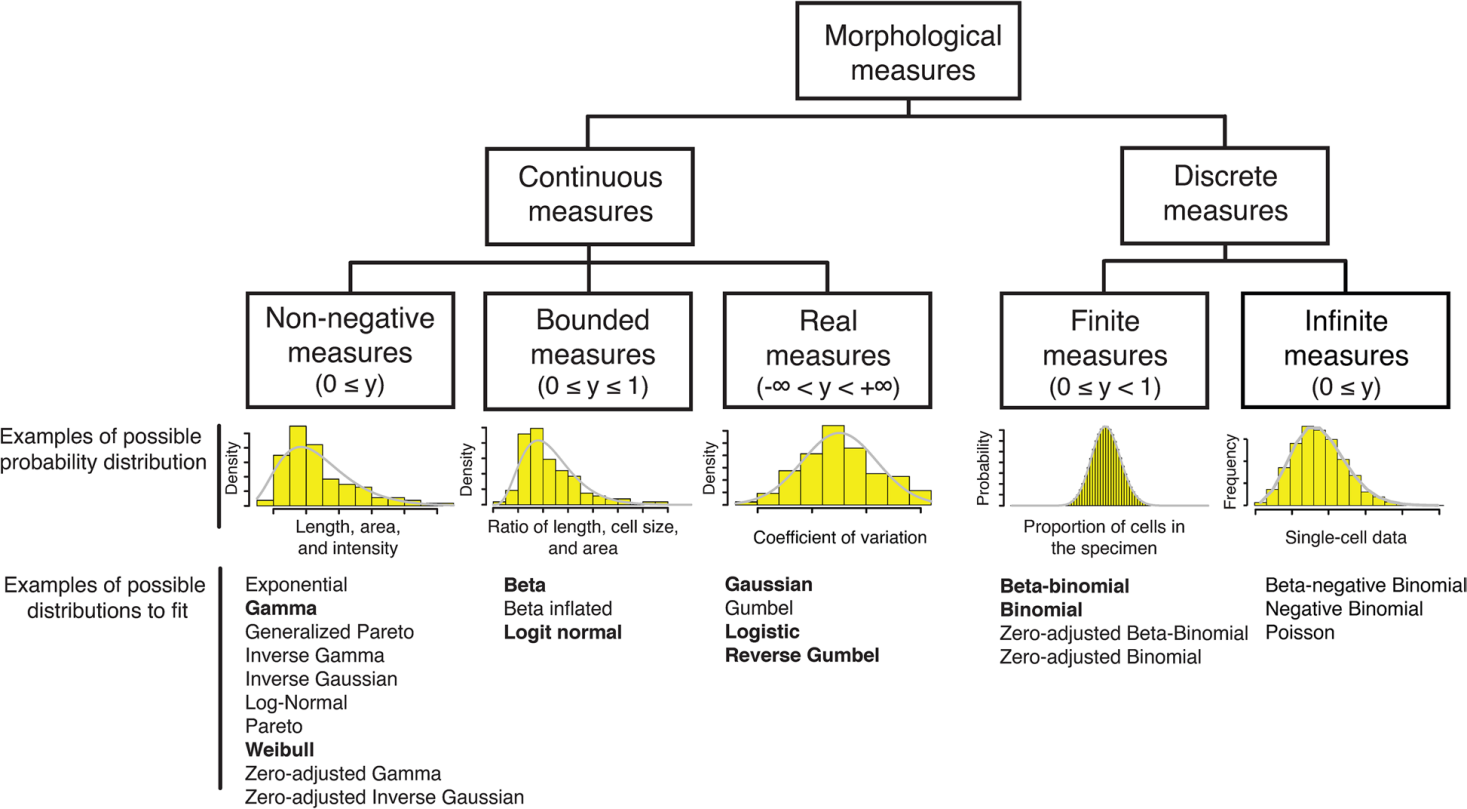
Various data types used for quantitative morphological phenotyping. Morphological measures were divided into five types. Continuous non-negative, bounded, real, and discrete finite measures were found in population-level studies, whereas discrete infinite measures were found in single-cell studies. Each type has a clear definition and distinct characteristics. Example possible distributions are sorted alphabetically. Bold font indicates the best-fitted distributions in this study. For a detailed list, see Supplementary Table S1

The features defined in cell morphology include the size, density, number, length, distance, and angle (Supplementary Fig. S1A). In addition, biological features associated with cell morphology include comparison measurements obtained, for example, by calculating ratios between two related morphological measurements, measuring variations in cell morphology, and comparing proportions of specific types of cells in the population (Supplementary Fig. S1B). The shapes and patterns of natural organisms (Fig. 2A) do not always follow the complete Gaussian distribution (Fig. 2B). Therefore, to anticipate precise and accurate values in morphometrics, we must define the best probability distribution in each scale (Fig. 3).

Many types of morphological measurement data [22], such as cell size, brightness, and length, and gradient-based image descriptors used in the field of computer vision [23], represent continuous non-negative measures (0 ≤ *y*; Fig. 3), generally showing skewed distributions. Of the 501 parameters used in the yeast CalMorph system, 183 parameters are non-negative values with skewed shapes where gamma and Weibull distributions fit well; 177 and 6 parameters, respectively, based on AIC model selection (Supplementary Fig. S2A; Supplementary Table S1A).

Ratio measurements, such as the axis ratio, represent the second data type, continuous bounded values between zero and one (0 ≤ *y* ≤ 1; Fig. 3), and are also mainly skewed-like beta distributions of the first kind (hereafter beta distribution). Among the CalMorph parameters, 37 parameters are expressed as ratios and can follow the beta (36 parameters) and logit-normal (one parameter) distributions (Supplementary Fig. S2B; Supplementary Table S1B).

Morphological noise can be defined by uncoupling the dependency between the coefficient of variation (CV) and the mean values [24, 25]. Continuous positive and negative real values can be obtained (−∞ < *y* < ∞) (Fig. 3) since the data are transformed around the locally estimated scatterplot smoothing fitted curve at zero (Supplementary Fig. S3A). As a result, the noise values can potentially generate a Gaussian distribution. There are 220 noise parameters in the CalMorph system, and the Shapiro–Wilk normality test revealed that 209 parameters are Gaussian distributed (*P* < 0.05, after Bonferroni correction; Supplementary Fig. S3B; Supplementary Table S1C). Further statistical analysis with the AIC revealed that among the remaining 11 parameters, five and six parameters fit logistic and type I extreme value (i.e., reverse Gumbel) distributions, respectively (Supplementary Fig. S2C; Supplementary Table S1C).

The proportions of discrete count data, such as the proportion of a specific type of cells, take finite values between zero and one (0 ≤ *y* < 1; Fig. 3). This type of data can follow either binomial or beta-binomial distributions, depending on the constantness of the stochastic event. There are 61 proportion parameters in the CalMorph system. To know which distribution fits the morphological dataset better, we performed analysis with the AIC and determined that 38 and 23 CalMorph parameters follow beta-binomial and binomial distributions, respectively (Supplementary Fig. S2D; Supplementary Table S1D).

We employed the AIC to show that the reference distributions identified previously [14], including gamma, beta, Gaussian, and binomial distributions for 177, 36, 209, and 23 CalMorph parameters, respectively, are better fitted than all other distributions (Supplementary Table S1). Thus, by defining the data type, followed by validation with the model selection method, the morphological parameters of CalMorph consist of four data types that can be defined by nine distributions, namely, gamma, Weibull, beta, logit-normal, Gaussian, logistic, reverse Gumbel, beta-binomial, and binomial distributions (Supplementary Table S1).

### Data modality

The modality of the distribution refers to how many modes exist in the distribution. It reflects the complexity of the distribution as well as the mixed populations generating the distribution. Therefore, a simple statistical approach can only be applied to the unimodal distribution [26]. To estimate the true values during QMP, unimodal distributions are preferable.

**Fig. 4:**
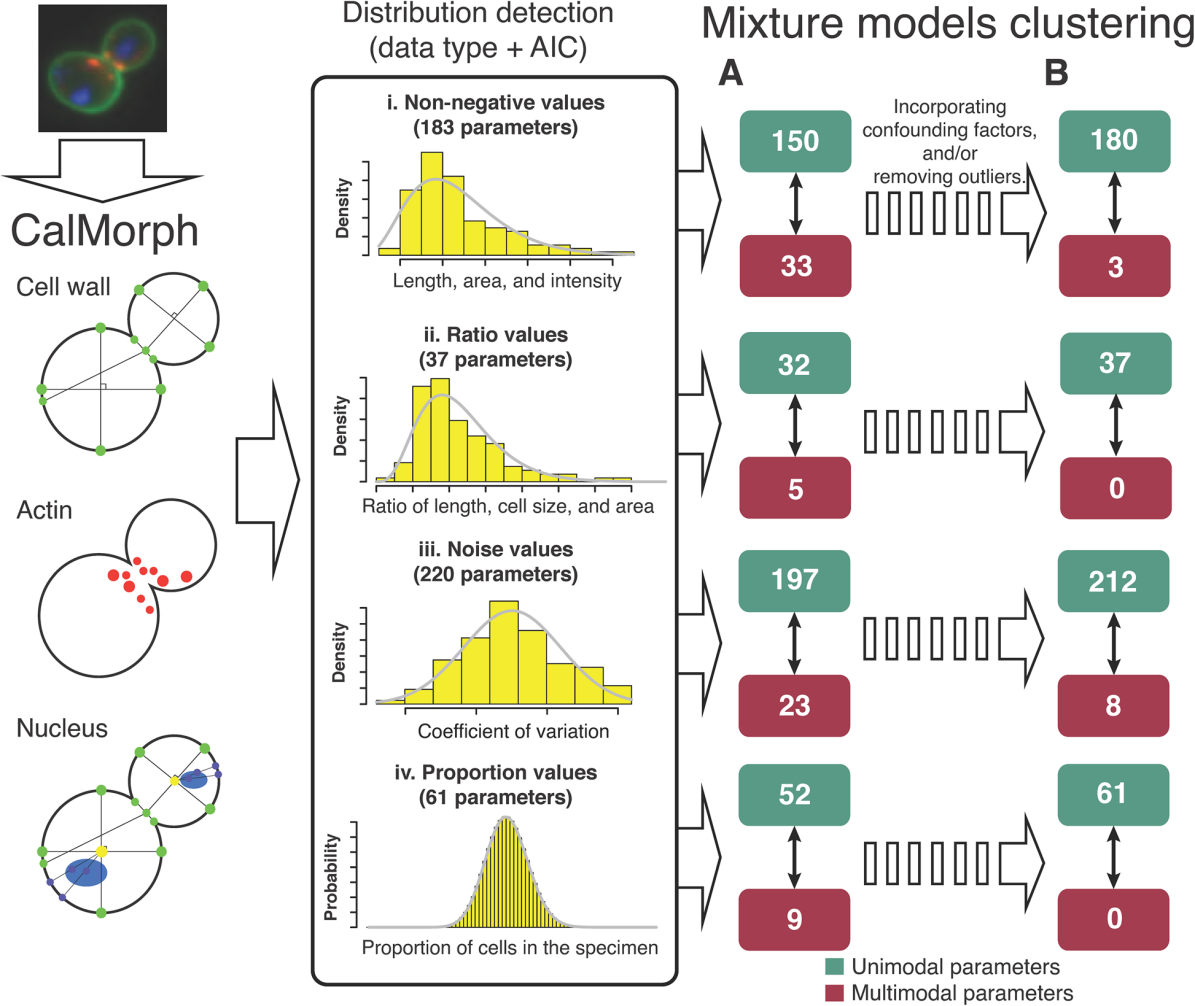
Modality analysis of the CalMorph morphological parameters. **A** Mixture-model-based clustering was used to check the modality of 501 CalMorph parameters according to the data types of (i) non-negative, (ii) ratio, (iii) noise, and (iv) proportion measures. Using 114 replicates of wild-type yeast cells revealed 431 unimodal and 70 multimodal parameters. **B** Confounding factors due to the differences in the experimental conditions and outliers were further considered as multimodal behaviors. A GLM was introduced by constructing a linear model (one-way analysis of variance) of the confounding factors for each parameter. Outlier data points were removed after defining a threshold for each parameter (one-percentile rule). As a result, 490 unimodal parameters were detected

To clarify the modality of the distribution for each morphological feature, we used a reliable and detailed set of experimental log data of wild-type morphological replicates. We also used a probabilistic mixture model to check the modality of the predefined probability distributions. We confirmed the most likely number of components (herein, we used 1 ≤ c ≤ 10) using the Bayesian information criterion (BIC; Supplementary Fig. S4) and iterations of randomization (see Supporting Text). Each of the 183 non-negative CalMorph parameters was subjected to gamma or Weibull mixture-model-based clustering. We found that 150 parameters showed a unimodal distribution. Likewise, 32, 197, and 52 parameters were unimodal out of 37 ratio, 220 noise, and 61 proportion parameters, respectively. In total, 431 (86%) of the 501 CalMorph parameters were unimodal (Fig. 4A; Supplementary Table S2).

There were still 70 (14%) CalMorph parameters whose distributions were multimodal. Because multimodality of a distribution can be caused by confounding factors and outliers, we investigated these possibilities in detail. We considered a group of five confounding factors, including a combination of different filters for microscopy and the image acquisition period, based on experimental logs (Fig. S5, inset). We introduced a generalized linear model (GLM), given the probability distribution for each parameter (Supplementary Table S1), of the confounding factors (Supplementary Fig. S5) and examined the multimodality again. In addition, we removed outlier data points after defining a threshold (one-percentile rule; Supplementary Fig. S6). As a result, the cumulative unimodal parameters summed up to 180, 37, 212, and 61 for non-negative, ratio, noise, and proportion parameters, respectively (Fig. 4B), increasing the unimodal frequency to 97.8% (490 out of 501). Thus, most of the CalMorph parameters exhibited unimodal distributions that can be used as the basic tools for further statistical analyses. Compared with the Box–Cox power transformation used in our previous study [9], UNIMO offers many more morphological parameters (Supplementary Fig. S7, Supplementary Table S3).

### Application of the proposed pipeline

**Fig. 5:**
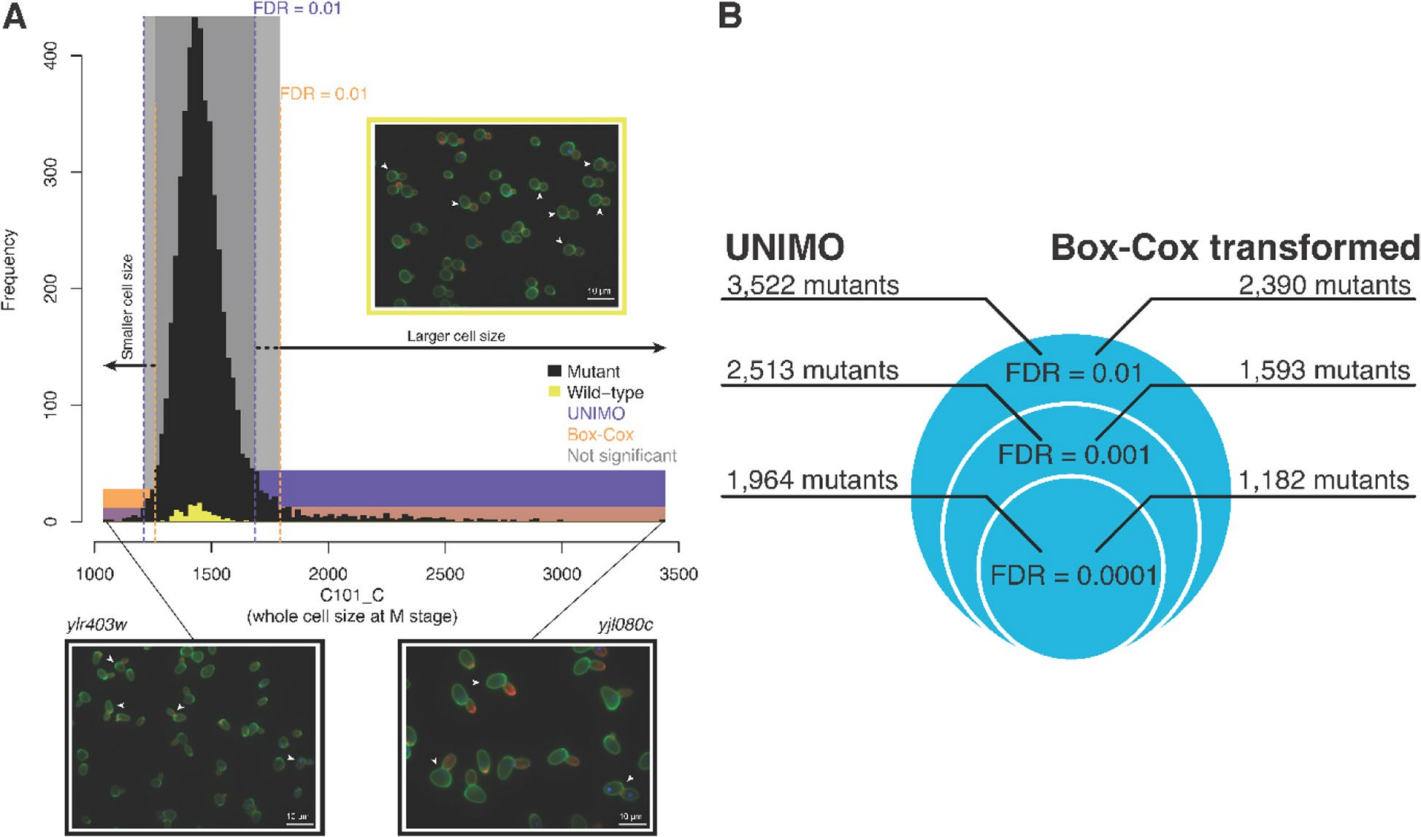
Morphological phenotyping of yeast non-essential mutants. **A** Histograms showing cell sizes at the M stage (C101_C); 4708 mutants and 109 *his3* replicates are shown in black and yellow, respectively. Dashed lines indicate significant thresholds for the UNIMO (purple) and Box–Cox transformed (orange) methods at FDR = 0.01. Example microscopy images of yeast cells are shown: actin, cell wall, and nucleus are shown in red, green, and blue, respectively. White arrows point to cells detected in the M stage by CalMorph. The unit for size was the number of pixels squared; for details, see the CalMorph user manual. **B** Yeast non-essential genes important for morphology are identified by applying UNIMO and Box–Cox transformation

To assess the power of the proposed UNIMO pipeline, we determined the number of morphologically distinct mutants by analyzing morphological changes of the 4708 haploid non-essential yeast mutants [9]. Employing 490 unimodal CalMorph parameters, we found that a total of 3522 mutant strains exhibited differences from wild-type cells in at least one parameter (Fig. 5; false discovery rate [FDR] = 0.01; Supplementary Table S4). Compared with our previous analysis [9] in which we found 2390 mutants with abnormal phenotypes, this study showed ~1.5 times greater power (FDR = 0.01; Supplementary Fig. S8). We also estimated the rate of false positives, which confirmed that the number of abnormal mutants was not overestimated in this analysis. These analyses suggested that our approach is precise enough to capture subtle morphological changes.

**Fig. 6:**
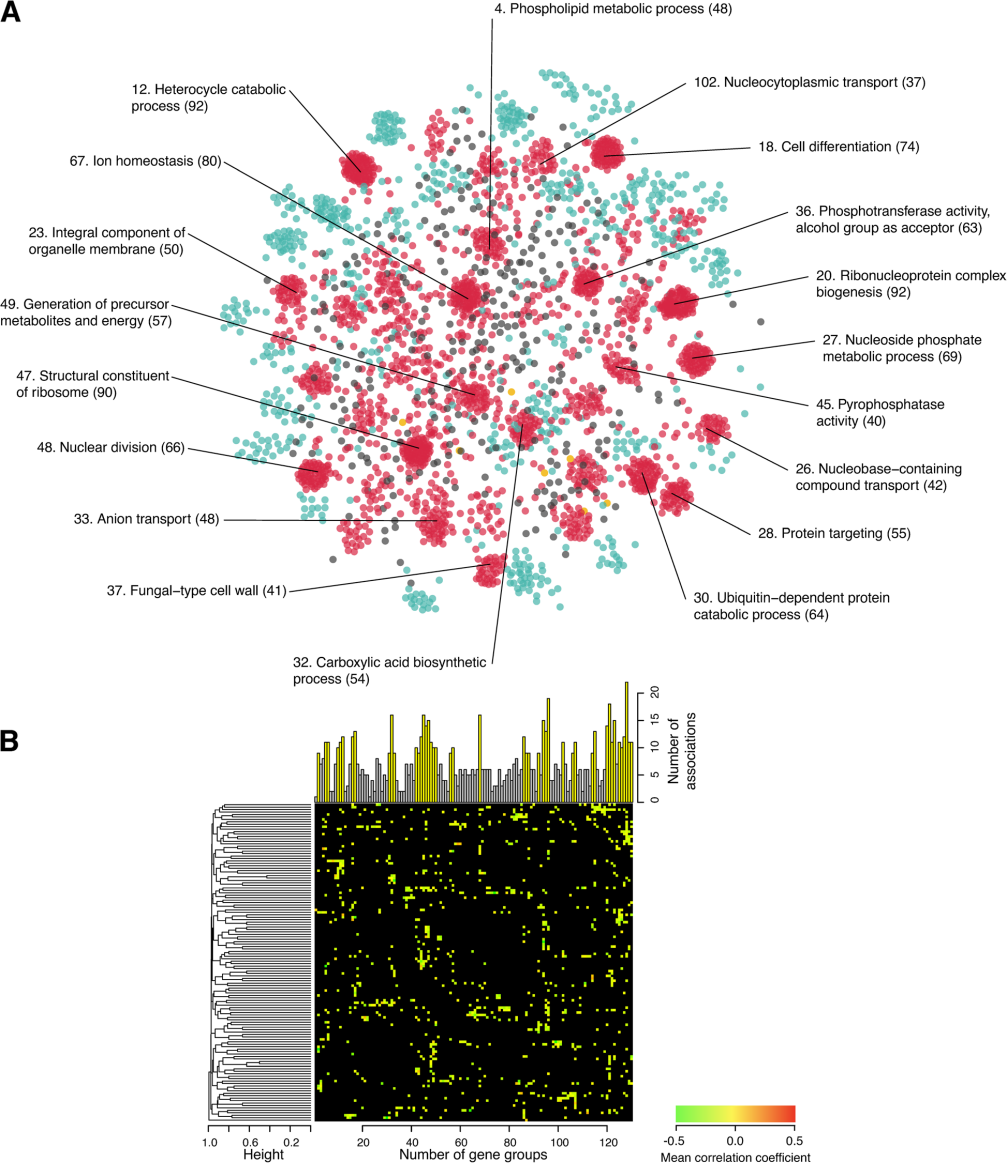
Overview of functional relationships between nonessential yeast genes. **A** Graphical representation of 2915 nonessential yeast genes with morphological defects and their functions is shown using the Spring layout. This network shows the similarities in phenotypes between pairs, calculated using 32 pCV scores and expressed as correlation coefficients (see the “Methods” section). Of the 130 functional groups (*P* < 0.05, after Bonferroni correction; Supplementary Table S5), 45 (orange and red dots) and 83 (turquoise and red dots) were identified as core and dense groups, respectively. Additionally, 44 groups (red dots) were identified as both core and dense groups. Of these 44 groups, most related GO terms belonged to 19 groups. Numbers in parentheses represent the number of group members. **B** Pairwise phenotypic correlation coefficients between functional gene groups. Significant correlations are shown as colored cells. Black cells indicate no significant correlation. The dendrogram was generated based on the proportion of significant correlations. The bar plot indicates the number of pairs with significant similarity. The 44 core and dense groups are shown in yellow (Supplementary Table S5)

Next, we investigated how important biological information can be obtained by using the UNIMO pipeline in combination with canonical correlation analysis (CCA). CCA was used to explore the relationships between two multivariate datasets (in this case, morphological phenotypes and gene functions). We compressed all combinations of 490 CalMorph parameters and 3127 Gene Ontology (GO) terms into linear combinations of phenotypic (32 phenotype canonical variables [pCVs]) and gene function (32 gene canonical variables [gCVs]) features (*P* < 0.05; Supplementary Fig. S9; see the “Methods” section). We used the correlations between morphological canonical variables to construct global functional maps of the non-essential yeast genes. Based on the relationships, we systematically mapped 2915 non-essential genes belonging to 130 GO terms (Supplementary Table S5). We observed 44 core gene groups containing 1900 non-essential genes with various functions, such as ion homeostasis (GO:0050801; 80 members), structural constituents of ribosome (GO:0003735; 90 members), generation of precursor metabolites and energy (GO:0006091; 57 members), and carboxylic acid biosynthetic processes (GO:0046394; 54 members); these served as hubs in the decentralized network and showed more significant associations than the others (Fig. 6A and Supplementary Fig. S10A). When the dendrogram was constructed on the basis of the proportion of significant correlations, core gene groups were not clustered (Fig. 6B and Supplementary Fig. S10), implying that even gene groups with a small number of associations play important and diverse roles through their interconnections. Our analyses using UNIMO and CCA provided an overview of the functional relationships between large numbers of non-essential genes based on morphological phenotypes.

We also explored the cellular pathways to evaluate the effect of the additional biological information obtained by the UNIMO pipeline. The Kyoto Encyclopedia of Genes and Genomes (KEGG) pathway enrichment analysis, using significantly abnormal mutants identified by the UNIMO method (*n* = 3522; FDR = 0.05), included more categories compared to the Box–Cox method (*n* = 2390; FDR = 0.05), such as metabolic pathways, autophagy processes, and protein processing (Supplementary Fig. S11 and Supplementary Table S6). This indicates the importance of the additional information obtained by UNIMO.

We mapped the newly detected cellular pathway by UNIMO to the global functional maps and found that the map contained information at multiple resolutions. For instance, we mapped 32 autophagy genes (Group 73; Supplementary Table S5) on the functional map at a higher resolution (Supplementary Fig. S12A), which enabled the detection of common morphological defects (Supplementary Fig. S12B). We found that *atg17∆* cells were the most defective cells, exhibiting significant differences in 90 morphological parameters (Supplementary Table S4). Among the *atg* mutants in this group, the morphology of *atg11∆* cells were similar to that of the *ATG17* knockout cells (*r* = 0.48, two-sided *t* test, *P* < 0.01). We further investigated the morphological similarities between *atg11∆* and *atg17∆* mutants (Supplementary Fig. S12C) in which gene deletions were characterized by altered mother cell shapes (C115_A, C115_A1B, C103_A, and C103_A1B), larger bud size (C11-2_C and C12-2_C), altered bud shape (C107_C), and altered nuclear morphology (D134_C, D185_C, and D186_C). Cell shape (C115_A; whole-cell axis ratio at the G1 stage) was similarly elongated in 20 mutants (green nodes in Supplementary Fig. S12B), indicating that these *atg* mutants had common morphological characteristics.

We successfully applied UNIMO to an archive morphological dataset of yeast non-essential genes and detected subtle morphological abnormalities that were ignored in our previous study [9]. Visualizing the global functional network and pathways enrichment analysis also provided novel biological insights.

## Discussion

The current study presents the reliable UNIMO pipeline for understanding the true values of morphological measures by assigning nine unimodal distributions to their probability distributions. After possible distributions were assigned to the morphological measures, the distribution models were validated with the AIC. We succeeded in preparing unimodal probability distributions for most of the yeast morphological features to ensure parametric analysis is applied to obtain biologically important information in the downstream analyses. This was conventionally overlooked previously, which can actually confine the power to distinguish morphological variations. Multimodality may instead lead to misinterpretation of the observed phenomena and affect the reproducibility of the results at certain points.

### Advantages of using unimodal probability distributions

Once the unimodal probability distribution models are defined for the morphological measures, any appropriate parametric methods can be employed in the downstream analyses. Application of parametric approaches allows for sensitive morphological distinctions: we used a relevant approach to identify more than half of the essential yeast genes as morphological haploinsufficient genes [14]. Another advantage is that we can use the GLM, an extension of the normal linear model [27], for hypothesis testing. Furthermore, we can employ various machine learning approaches to recognize, predict, understand, and obtain data/knowledge. In this way, parametric approaches can be applied in the future to perform correlation analyses to compare morphologies [28], classification analyses to distinguish categories based on morphology [29], prediction analyses to identify similar morphologies [30], factor analyses to explore potential common factors [31], analysis of morphological diversity for breeding purposes [32, 33], and analysis of sources of bias in microfluidic cell culture research [34].

### Various probability distributions fitted to the morphological data

We showed that our QMP data contained four different data types that can well be explained by nine distributions. Defining the best probability distribution model is one of the most important factors in this study. However, we believe that the same nine probability distributions may not always be selected during QMP. For example, non-negative values may form heavily skewed probability distributions (e.g., inverse Gaussian). At the single-cell level, Poisson or negative-binomial distributions are commonly used for count analyses. Eventually, for non-negative, ratio, and proportion measures, various inflated distributions (zero-, one-, or both, according to the distribution) should also be considered to accommodate special cases in which there is a mixture of discrete values and a continuous distribution. Thus, while this study provides a solid foundation for phenome studies, researchers may need to identify the best-fitting model to accommodate specific morphological measures.

### Unimodal distribution of the morphological measures

Data modality is another concept that we took into account in this study. Since simple explanations are preferred until the data justify a complex model, a unimodal distribution is better than a multimodal distribution a priori. This is the principle of Occam’s razor that entities should not be multiplied needlessly. But more practically, a unimodal probability distribution is better because it can be employed for parametric analysis, providing a simple assumption to detect significant changes and improve accurate interpretation of the results.

We found that most of the probability distributions of the yeast morphological features are unimodal. However, unimodal features may not be always prepared. The reason why this study was successful is that many single-cell parameters of CalMorph exhibited unimodality [35], according to the automatic classification of cells based on the cell cycle stage [9]. We believe that an important benchmark is the number of unimodal, single cell parameters when applying UNIMO to any image processing tool. In the case of multimodality, we can use distinct cell attributes, such as the cell cycle stage [9], cell shape [16], and cell type [36, 37]. Furthermore, it is essential to consider the effects of confounding factors. The 11 remaining multimodal parameters (Supplementary Fig. S13) may be either unknown confounders or multimodal as a property of the morphological features in the first place.

Finally, to illustrate the impact of this study, we thought it might be possible to apply UNIMO to archived imaging data. However, affirming this is challenging because labs do not publicly share morphological measures of standard data sets with experimental logs alongside the perturbed cells. The Supporting Text, Supplementary Fig. S14, and Supplementary Table S8 elaborate on the versatility of this pipeline using a hypothetical standard data set of morphological measures obtained by CellProfiler [12] and using data from [38]; Supplementary Fig. S15 and Supplementary Table S9 show the results.

## Conclusion

The estimation of true values is the ultimate goal in quantitative morphological analyses. Due to its simple mathematical and computational specifications, biologists tend to first try the Gaussian distribution, although morphological features are not always normally distributed. Non-normal data are transformed to obtain approximately normal distributions, but the first choice after normalization failure is a non-parametric method. Nevertheless, to detect subtle differences, higher statistical power is desirable; therefore, the application of parametric approaches allows for clearer morphological distinctions. In this study, we demonstrated a better prediction method of the true values of morphological measurements using UNIMO. We also demonstrated how these concepts can provide biologically important information. Our study offers a framework for future phenome studies and enables further development of a typical QMP pipeline.

## Methods

### Selection of the probability distribution for each morphological measure

We determined models of the probability distributions for each of the 501 morphological parameters to accommodate the statistical model used in the GLM [20]. The AIC was employed to find the best-fitted model. Figure 3 and Supplementary Fig. S1 present the logic behind the procedure. More details are also described in the Supporting Text.

### Population level modality check

We used mixture-model-based clustering [39, 40] to check the modality of 501 CalMorph parameters according to predefined distributions (Supplementary Table S1). The BIC was used to compare the primary probability models that differ in the number of components (*c*); a mixture of 1 ≤ *c* ≤ 10 distributions was tested. Supplementary Fig. S4 illustrates a flowchart of the modality check. Further explanations are provided in the Supporting Text.

### Tracking morphological variations in the non-essential gene mutants using the proposed pipeline

To test the effectiveness of the proposed pipeline, we reanalyzed morphological variations in the 4718 non-essential yeast gene mutants [9]. Of the 4718 open reading frames (ORFs), 4 (YAR037W, YAR040C, YGL154W, and YAR043C) were deleted from the *Saccharomyces* Genome Database (SGD; https://www.yeastgenome.org/), and 6 were merged with other ORFs (Supplementary Table S4). Ultimately, we compared 4708 mutants to a dataset of haploid wild-type yeast strains (*his3*; 109 replicates [41]) using UNIMO. Models of the probability distributions of the 490 UNIMO parameters were used to accommodate the pre-defined statistical models (Supplementary Table S3). We calculated the P value as the deviation of each mutant from the wild-type strains based on the estimated two-tailed probability distribution using the distribution probability functions in the gamlss package (e.g., pGA, pBE, pNO, and pBI functions). For each function, the P value was calculated from the mean and dispersion (if applicable; for example, binomial distribution does not consider dispersion) of the model fitted to the wild-type population. The FDR, i.e., the rate of type I errors for the rejected null hypothesis due to multiple comparisons, was estimated using the q value function in the q value package [42]. Ultimately, we estimated the number of mutants with significant changes, if any, and abnormal morphology in at least one parameter (FDR = 0.01).

### Z value transformation

To estimate Z values, after the maximum likelihood estimation (MLE) had converged, morphological data were transformed to Z values using the Wald-test (one-sample two-sided test using the summary.gamlss function of the gamlss package), where the Z value in the *i*th parameter of the *j*th mutant is [20]:

$${Z}_{ij}=\frac{\beta_{ij}-{\beta}_{i0}}{SE_{ij}}$$

Here, *β*_*ij*_ is the MLE of the *j*th mutant, *β*_*i0*_ is the MLE of the null distribution (109 replicates of the haploid wild-type yeast strains), and *SE*_*ij*_ is the standard error. For the beta-binomial distributed parameters (Supplementary Table S3D), if the numerator of a mutant was equal to estimated value from the wild-type population, the Z value was set to zero; otherwise, it was set to the maximum value of the other mutants.

### Construction of the morphological-functional network

#### Gene Ontology annotation

For the GO annotation analysis, we downloaded the basic version of the GO file from the GO Consortium (http://geneontology.org/) and gene annotations from the SGD. A Boolean matrix of GO terms of 3522 genes with abnormal morphologies (UNIMO; FDR = 0.01) was generated as the functional profile (if a gene was annotated by a GO, the value was 1; otherwise, it was 0). We selected 3127 GO terms, each of which was annotated for more than two genes (i.e., removing unique terms) and less than 200 genes (i.e., removing global terms) in the genome with no identical sets of annotated genes. We excluded 577 genes that were not annotated by the 3127 GO terms (i.e., removing genes with no functional reports).

#### Canonical correlation analysis

We used CCA to reduce the dimensions from 490 parameters and identify biologically important morphological features. For this purpose, we used the Z values of 2945 genes as the morphometric profiles (i.e., 2945 × 490) and the Boolean matrix of GO terms as the functional profile (i.e., 2945 × 3127). To reduce the dimensionality, we subjected the morphometric profiles to principal component analysis (PCA; prcomp function of the R stats package). The initial 146 PCs (hereinafter referred to as phenotype principal components; pPCs) accounted for more than 95% of the data variation (i.e., the cumulative contribution ratio; CCR). Next, to estimate the functional relationships among the 2945 genes, we used the structure of 3127 GO terms. The dimensionality of the functional profiles was reduced by PCA according to [43], with some modifications. Briefly, we applied PCA on the Boolean matrix of all genes (i.e., 2945 × 3127). This approach reduces the dimensionality while preserving the structure of the functional relationships among the genes. The first 1474 PCs (hereinafter referred to as GO term principal components; gPCs) explained 99% of the data variation, indicating that approximately 1474 gene functions were related to the 2945 genes.

After projection of the Z values on pPCs and a zero matrix on gPCs for 109 replicates of the wild-type population, we applied CCA to 146 pPCs (CCR = 95%) and 1474 gPCs (CCR = 99%). The significance of the canonical correlation coefficients was tested using Bartlett’s chi-squared test with the significance level set at *P* < 0.05 [44]. Ultimately, 32 morphological features (pCVs) and 32 gene function features (gCVs) were obtained (Supplementary Fig. S9).

#### Pairwise canonical correlation analysis

We divided the genes into functional groups with no common term. The 2945 nonessential genes with at least one GO annotation by the 3127 GO terms were clustered into disjunctional functional gene groups using common GO annotations. The binary distance between each pair of genes was calculated on the basis of a Boolean matrix of 3127 GO terms and applied for hierarchical cluster analysis by the complete linkage method (i.e., minimum ratio of the different genes between clusters; hclust R function). Using “static branch cutting” at a height < 1, 150 gene groups were identified, each of which contained 1–92 genes. We selected groups with at least two members (130 groups). To assign the most appropriate GO terms to each gene group, enrichment of the GO terms was analyzed using Fisher’s exact test (P < 0.05, after Bonferroni correction). In 122 of the 130 groups, more than one GO term was enriched. The remaining groups were therefore identified as functional gene groups with no GO terms in common. For each of the 122 groups, the GO term detected using the lowest P value was selected as the representative function (Supplementary Table S5).

Next, we calculated pairwise correlation coefficients between the functional gene groups. To detect significant relationships between the gene groups, we performed pairwise CCA between arbitrary pairs of 130 gene groups (_130_C_2_ = 8385) using 32 pCV scores. To prevent detection bias, we used a smaller number of genes than the number of pCVs by reducing dimensionality of the genes after applying PCA to the data of nonessential genes. For pairwise CCA, we applied CCA to pCV scores using genes and/or selected PCs as variables and extracted canonical variables of the gene deletion mutants (mCVs) as independent components with correlations between gene groups. We tested the significance of the canonical correlation coefficient of the first mCV at *P* < 0.0005, after Bonferroni correction using Bartlett’s chi-squared test [44]. Among 8385 pairs in 130 gene groups, 472 had significant relationships between groups.

### Visualization of the phenotypic correlations

The similarity of phenotypes between pairs (among the 2945 nonessential gene deletion mutants) was calculated using 32 pCV scores and expressed as correlation coefficients (*r*). To visualize the network of the 122 GO term-enriched gene groups (2890 genes) with significant relationships to other groups, we used the qgraph package and Spring layout [45]. We populated the matrix of pCV score-based correlation coefficients after zero-filling cells using the qgraph function, when no significant correlation was detected in mCV1 between groups or the absolute value of the correlation coefficient between genes of different groups was not the maximum.

#### Core gene groups

We identified core gene groups with significant correlations. We divided the groups into two clusters by applying Poisson mixture-model-based clustering and identified groups having significant correlations with nine or more other groups as core groups (Supplementary Fig. S16A).

#### Dense gene groups

We identified dense gene groups, in which the genes were closer to each other in the correlation network. We divided the groups into two clusters by applying gamma mixture-model-based clustering to the average distance between arbitrary pairs of genes in the two-dimensional network and identified the groups with an average distance between genes of < 0.2 as dense groups (Supplementary Fig. S16B).

#### Pathway enrichment

Functional categories of significant ORFs detected by the UNIMO and Box–Cox transformation methods (3522 and 2390 ORFs, respectively) were enriched in the KEGG database using clusterProfiler package [46] at FDR = 0.05.

## Supplementary Information

**Additional file 1: **Supplementary text. **Supplementary Figure S1.** Possible values of cellular features used in QMP. **Supplementary Figure S2.** Probability models for CalMorph measures. **Supplementary Figure S3.** Conversion of the CV to noise values. **Supplementary Figure S4.** Flowchart of the methodology for checking the modality of the CalMorph parameters. **Supplementary Figure S5.** An example of the effect of confounding factors on modality. **Supplementary Figure S6.** An example of the effect of outliers on modality. **Supplementary Figure S7.** Comparison of this study with our previous results. **Supplementary Figure S8.** Comparison of results between UNIMO and Box-Cox transformed methods. **Supplementary Figure S9.** Canonical correlation analysis used for extraction of 32 pairs of canonical variables. **Supplementary Figure S10.** Phenotypic similarity network of non-essential genes. **Supplementary Figure S11.** Enrichment of KEGG categories. **Supplementary Figure S12.** Morphological defects of autophagy mutants. **Supplementary Figure S13.** Multimodal CalMorph parameters. **Supplementary Figure S14.** Outlines of generalization of UNIMO. **Supplementary Figure S15.** An example of versatility of UNIMO. **Supplementary Figure S16.** Defining characteristics of the 130 functional groups. **Supplementary Table S1.** Selection of the probability distribution for each morphological parameter; non-negative (A), ratio (B), noise (C), and proportion (D) measures. **Supplementary Table S2.** Population level modality check for each morphological parameter; non-negative (A), ratio (B), noise (C), and proportion (D) measures. **Supplementary Table S3.** The best probability distribution and final modality for each morphological parameter; non-negative (A), ratio (B), noise (C), and proportion (D) measures. **Supplementary Table S4.** List of non-essential mutants. **Supplementary Table S5.** List of the representative GO terms enriched in each functional group. **Supplementary Table S6.** KEGG pathway enrichment (FDR = 0.05). **Supplementary Table S7.** Data types and probability distributions employed for a typical quantitative morphological phenotyping (QMP) experiment. **Supplementary Table S8.** List of measurements extracted by CellProfiler. **Supplementary Table S9.** An example of generalization and versatility of UNIMO using morphological data presented in Mattiazzi Usaj et al. (2020).

## Data Availability

The CalMorph morphological data and microscopy images used for this study are publicly available at http://www.yeast.ib.k.u-tokyo.ac.jp/SCMD/index.php. All source codes are also available for download at GitHub: https://github.com/OhyaLab/UNIMO.
