# Supplementary material for: Assignment of unimodal probability distribution models for quantitative morphological phenotyping
Source: BMC Biol. 2022 Mar 31;20:81. doi: 10.1186/s12915-022-01283-6 (PMC8969357; doi:10.1186/s12915-022-01283-6)
Supplement: Supplementary file 1 — Additional file 1: Supplementary text. Supplementary Figure S1. Possible values of cellular features used in QMP. Supplementary Figure S2. Probability models for CalMorph measures. Supplementary Figure S3. Conversion of the CV to noise values. Supplementary Figure S4. Flowchart of the methodology for checking the modality of the CalMorph parameters. Supplementary Figure S5. An example of the effect of confounding factors on modality. Supplementary Figure S6. An example of the effect of outliers on modality. Supplementary Figure S7. Comparison of this study with our previous results. Supplementary Figure S8. Comparison of results between UNIMO and Box-Cox transformed methods. Supplementary Figure S9. Canonical correlation analysis used for extraction of 32 pairs of canonical variables. Supplementary Figure S10. Phenotypic similarity network of non-essential genes. Supplementary Figure S11. Enrichment of KEGG categories. Supplementary Figure S12. Morphological defects of autophagy mutants. Supplementary Figure S13. Multimodal CalMorph parameters. Supplementary Figure S14. Outlines of generalization of UNIMO. Supplementary Figure S15. An example of versatility of UNIMO. Supplementary Figure S16. Defining characteristics of the 130 functional groups. Supplementary Table S1. Selection of the probability distribution for each morphological parameter; non-negative (A), ratio (B), noise (C), and proportion (D) measures. Supplementary Table S2. Population level modality check for each morphological parameter; non-negative (A), ratio (B), noise (C), and proportion (D) measures. Supplementary Table S3. The best probability distribution and final modality for each morphological parameter; non-negative (A), ratio (B), noise (C), and proportion (D) measures. Supplementary Table S4. List of non-essential mutants. Supplementary Table S5. List of the representative GO terms enriched in each functional group. Supplementary Table S6. KEGG pathway enrichment (FDR = 0.05). Supplementary [file 12915_2022_1283_MOESM1_ESM.zip › Supporting Text_ESM.docx]

Supporting Text for

Assignment of unimodal probability distribution models for quantitative morphological phenotyping

Farzan Ghanegolmohammadi, Shinsuke Ohnuki, Yoshikazu Ohya^*^

^*^Address correspondence to: Yoshikazu Ohya (e-mail: [ohya@edu.k.u-tokyo.ac.jp](mailto:ohya@edu.k.u-tokyo.ac.jp)).

**Table of Contents**

Supplementary text 2

Selection of the probability distribution for each morphological parameter 2

Data type and specific probability distributions 2

Non-negative continuous values 2

Bounded continuous values 3

Real continuous values 3

Finite discrete values 4

Selecting the best fitted probability distribution 4

Population-level modality check 5

Distribution-based mixture modeling 5

Population-level quality control 5

Generalization and versatility of UNIMO 6

Visualization of the phenotypic correlations for Box-Cox method 8

Supplementary Figures 9

Supplementary Tables 25

References 26

# Supplementary text

All statistical analyses were performed in the R environment (<http://www.r-project.org)>. R codes and morphological data used for this study are publicly available at <https://github.com/OhyaLab/UNIMO> and <http://www.yeast.ib.k.u-tokyo.ac.jp/SCMD/index.php>, respectively. This study does not aim to provide a new R package but building on the available R packages which have been developed through extensive efforts. These R packages are “gamlss” [1] for distribution fitting, “gamlss.mx” [2] and “mclust” [3] for modality check.

## Selection of the probability distribution for each morphological parameter

In this study, we used quantified values of images processed by CalMorph, a software designed for budding yeast cells [4]. CalMorph quantifies microscopic images of triple-stained yeast cells (cell walls, nuclei, and actin cytoskeletons) into 501 parameters, including averages of size, shape, orientation, and the ratio of cells. A dataset of 114 diploid wild-type yeast strains (BY4743; 31,042 cells) was used to define the best-fit distribution(s).

Morphological parameters include a variety of measurements that can be categorized into five groups (Figure 3 and Supplementary Table S7). The complex mathematics of different distributions are translated into simple codes by the “gamlss” package [1]. These packages provide functions for fitting more than 20 probability distributions and their derivatives, including various continuous, discrete, and mixed distributions.

## Data type and specific probability distributions

Models of the probability distributions for each of the 501 morphological parameters were determined to accommodate the statistical model used in the generalized linear model (GLM) using the gamlss package.

### Non-negative continuous values

Morphological measurement data such as cell size, counts, brightness, length, *etc*. represent non-negative continuous values [0, +∞). Of the 501 CalMorph parameters, 183 parameters are non-negative values (*e.g.*, length, area, and intensity) and are assumed to be gamma-distributed as the reference distribution [measured value (*y* > 0), mean (*μ* > 0), and dispersion (*σ* > 0)] as follows:

$$GA\left( y | \mu,\sigma\right)=\frac{y^{1/\sigma^{2}-1}e^{-y/\left( \sigma^{2}\mu\right)}}{\left( \sigma^{2}\mu\right)^{1/\sigma^{2}}\Gamma\left( 1/\sigma^{2} \right)}$$

The link function for gamma-distributed parameters is $f(y) =log(y)$. To check the equality of the data with the presumed reference distribution (*i.e.*, gamma), we compared each non-negative parameter to the reference probability distribution [generated by the pGA() function in R using the mu and sigma of each parameter] using the Kolmogorov–Smirnov test [the built-in ks.test() function in R]. We also tested another 14 probability distributions to thoroughly check possible exponential and negative skewedness behaviors of the data as well as special cases when the data contained zero values; that is, a mixture of discrete values (“0”) and a continuous distribution (Supplementary Table S7).

### Bounded continuous values

The ratio of each measurement is a bounded, skewed-like beta-distribution of the first kind. Of the 501 CalMorph parameters, 37 parameters range from zero to one; for example, the ratio of length, ratio of cell size, and ratio of area [measured value (0 < *y* < 1), mean (0 < *μ* < 1), and dispersion (0 < *σ* < 1)] as follows:

$$BE\left( y | \mu, \sigma\right)=\frac{1}{B\left( \alpha,\beta\right)}y^{\alpha-1}(1-y)^{\beta-1} where \left\{ \begin{aligned} \alpha=\mu\left( 1-\sigma^{2} \right){/\sigma}^{2} \\ \beta=(1-\mu)\left( 1-\sigma^{2} \right){/\sigma}^{2} \end{aligned} \right.$$

The link function for beta-distributed parameters is $f(y) = log[y/(1-y)]$. To check the equality of the data with the presumed reference distribution (*i.e.*, beta), we compared each ratio parameter to the reference probability distribution [generated by the pBE() function in R using the mu and sigma of each parameter] using the Kolmogorov–Smirnov test [the built-in ks.test() function in R]. We additionally checked another six probability distributions to research specific cases where the data are a mixture of discrete values (only “0” or “1”, or both) and continuous beta-distributed values (Supplementary Table S7).

### Real continuous values

Of the 501 CalMorph parameters, 220 parameters are the coefficient of variation (CV) of their related mean values. CV parameters are non-linearly dependent on the mean values [5]. To uncouple this concomitant dependency, LOESS (locally estimated scatterplot smoothing) regression with a smooth span is a proper approach. However, the best smooth span might be challenging. Here, we employed the gamlss() function as follows:

$$gamlss(y\sim lo(\sim x, span=f), data=data, family=NO)$$

where *y* is a CV parameter, and *x* is its related mean parameter and *f* is the smooth span of the LOESS regression (values ranging 0.10 ≤ *f* ≤ 0.99). We then used the Akaike information criterion (AIC) to choose the best-fit model. Finally, instead of CV parameters, noise parameters as the residuals (*i.e.*, observed value − predicted value) are calculated [6].

Noise parameters are assumed to be normally distributed [measured value (−∞ < *y* < ∞), mean (−∞ < *μ* < ∞), and dispersion (*σ* > 0)] as follows:

$$NO\left( y | \mu, \sigma\right)=\frac{1}{\sqrt{2\pi}\sigma}\exp\left[ -\frac{(y-\mu)^{2}}{{2\sigma}^{2}} \right]$$

The Link function for Gaussian-distributed parameters is $f(y) = y$. We checked the normality assumption using the Shapiro–Wilk normality test [the built-in shapiro.test() function in R]. Moreover, we checked another four probability distributions to survey moderate changes of skewness and kurtosis in only non-normal parameters (Supplementary Table S7).

### Finite discrete values

Proportions of discrete count data are bounded values between zero and one [0,1). Of the 501 CalMorph parameters, 61 parameters are proportions of cells in the specimen, which are assumed to be binomially distributed [number of particular cells (*y* = 1, 2, …, n), number of all cells (*n* ≥ 0), and mean (0 < *μ* < 1)] as follows:

$$BI\left( y | n, \mu\right)=\frac{n!}{y!\left( n-y \right)!}\mu^{y}({1-\mu)}^{n-y}$$

The link function for binomially distributed parameters is $f(y) = log[y/(1-y)]$. To check the equality of the data with the presumed reference distribution (*i.e.*, binomial), we compared each proportion parameter to the reference probability distribution using the Kolmogorov–Smirnov test [the built-in ks.test() function in R] by generating 100-fold parametric bootstraps. We additionally checked if the probability of success in each trial was beta-distributed (*i.e.*, beta-binomial) and “zero” values were also presented in the data (Supplementary Table S7).

### Selecting the best fitted probability distribution

As stated above, considering the data range and variable type, we chose gamma, beta, Gaussian, and binomial as the reference distribution for groups one through four, respectively. Moreover, we selected other probability distributions to cover a wider range of plausible probability models to determine which model has the best predictive accuracy and fewer hyper-parameters; *i.e.*, as simple as possible but not the simpler model [7]. This is because the probability models with more hyper-parameters (*i.e.*, three or four) require higher computation power and run-time, and may also cause problems in model convergence, especially in large datasets. However, for mixed models (*i.e.*, mixture of discrete values and a continuous distribution), we disregarded the number of hyper-parameters in employing the probability model. We used mixed models when a given data type has boundaries; such as ratio values [0,1]. Eventually, we considered the AIC as the measure of parsimony:

$$AIC=2k-2ln(L)$$

where, *k* is the number of hyper-parameters in the model and *L* represents the maximum value of the likelihood function for the model.

We selected one of the alternative distributions over the reference distribution only if the AIC of the alternative distribution was less than the reference distribution and:

$$\left| {AIC}_{ref}-{AIC}_{A} \right|> 10$$

where *ref* is the reference distribution and *A* represents all other alternative distributions defined based on the data type and range (Supplementary Table S7). Note that this criterion was not applied to the noise parameters; noise parameter alternative distributions were considered only for non-normal parameters (when the Shapiro–Wilk normality test was significant; P value < 0.05 after Bonferroni correction).

## Population-level modality check

The modality of each parameter was checked according to its pre-defined distribution. Supplementary Figure S4 presents a flowchart of the procedure. Also, to check if the observed uni-/multi-modality is obtained by chance, a Poisson distribution, generated by 3000 iterations of randomization of 50% of the data (n = 57), was used to estimate the P value.

### Distribution-based mixture modeling

We used mixture-model-based clustering, a flexible parametric method [8], to check the modality of the 501 CalMorph parameters according to their designated distributions (Supplementary Table S1). The Bayesian approach is potentially more informative, especially when the number of components is unknown [8]. We used the gamlss.mx package [2], ver. 4.3-5, for all probability distributions except Gaussian. For the Gaussian-distributed parameters, we used the mclust package (ver. 5.4.5) given that univariate data have equal volumes [3]. In all cases, we used the Bayesian information criterion (BIC) to compare the primary probability models that differed in the number of components:

$$BIC=ln\left( n \right)k-2ln(\hat{L})$$

where, *n* shows the number of observations (*i.e.*, sample size), *k* is the number of hyper-parameters in the model multiplied by the number of the clusters (a mixture of 1 ≤ *c* ≤ 10 distributions was tested), and $\hat{L}$ represents the estimated maximum value of the likelihood function for the model.

### Population-level quality control

#### Confounding factors

To minimize the effects of experimental error among replicates [9], a group of five confounding factors was considered, including a combination of different fluorescence filters for microscopy (see below) and the period of image acquisition based on experimental logs, shown in Supplementary Figure S5 inset and explained in [10]. A GLM was introduced by constructing a linear model (one-way analysis of variance) of the confounding factors (confounding factor model; CFM):

$$f\left( y \right)=\beta_{1}MS1+\beta_{2}MS2+\beta_{3}MS2a+\beta_{4}MS2b+\beta_{5}MS3$$

where *y* is the fitted value, *f* is an appropriate link function, *MS1* is microscope #1 before replacement of the fluorescence filter, *MS2* is microscope #2 before replacement of the fluorescence filter, *MS2a* is microscope #2 after replacement of the fluorescence filter over time, *MS2b* is microscope #2 after replacement of the fluorescence filter, *MS3* is microscope #3, and *β* is the fixed effect of each confounding factor (0,1). The best-fitted model between the CFM and the null model was selected using the AIC.

#### Outliers

Highly unusual phenotypes might be observed due to errors in sample preparation, imaging, and image processing, such as incorrectly segmented cells, out-of-focus cells, and staining artifacts [9]. Considerable distances of the outliers might mislead the model in detecting individual distributions. In the case of edge peak distributions, a model-free outlier-detection method, the one-percentile deviation rule, was used to remove expected sets of outliers from the multimodal parameters (Supplementary Figure S6).

## Generalization and versatility of UNIMO

UNIMO can be applied to a wide range of morphological phenotyping studies (Supplementary Figure S14). For this aim, one needs to follow these steps:

1. Finding data type for each morphological measure by comprehending the meaning of each measure. See Figure 3 for better understanding. At the end of this step, one has to be able to make a table (data-type assignment table) in which rows are extracted morphological parameters and columns are “Characteristics of the data”, “Reference distribution”, and “Alternative distribution(s)”, like Supplementary Table S7. Supplementary Table S8 is an example of morphological measurements extracted by Cell Profiler.
2. Preparing a standard repeated experimental data set (standard data set) which can be regarded as the null distribution. We here used a data set of 114 diploid WT yeast strains (BY4743).
3. Defining the best probability distribution in each parameter by fitting applicable probability distribution to the standard data set. For this purpose, one can use data-type assignment table (Supplementary Table S7 or Supplementary Table S8). There are more distributions available in “gamlss” R package [1], but here we chose only those probability models which are well-known for each data type and less complex. Then, the best fit model was selected for each parameter in an object manner using AIC. The gamlss function (gamlss R package) estimates AIC for the fitted model. We here defined the best probability distribution in each CalMorph parameter (Supplementary Table S1). See *Selecting the best fitted probability distribution* section for AIC formula.
4. Performing population-level quality control by using the standard data set with experimental log data (standard data set with experimental log). Biological or technical factors may produce subpopulations (if there is any) causing data modality. In this study, we considered a group of five confounding factors, including a combination of different filters for microscopy and the image acquisition period, based on experimental logs (Supplementary Figure S5). We also considered outliers (Supplementary Figure S6).
5. Checking modality of each parameter given the best fitted probability distribution and possible confounders by using mixture-model-based clustering (see *Population-level quality control* section).

One always needs to revise scripts according to his/her own data in hand but our scripts at <https://github.com/OhyaLab/UNIMO> provide a clear foundation for other researchers to build on. Note that, prior to implementation of UNIMO, a dataset which meets the requirements mentioned in step 2 and 4 must be prepared. To the best of our knowledge, there is no publicly available dataset which meets those requirements. Generally, labs do not share morphological measures of standard data sets with experimental logs. This issue posed a serious problem to show the generalizability of UNIMO. However, to confirm the versatility of UNIMO, we chose mutants with no morphological defects (FDR = 10%; 138 mutants) in our study assuming that these mutants resemble morphology of the WT cells to certain extend. Then, we applied UNIMO to the morphological data of these mutants presented in [11] in which CellProfiler [12] was used for object segmentation and quantitative feature extraction and data are publicly available at <https://thecellvision.org/endocytosis/supplementalb>. We checked the best probability model (Supplementary Table S9A) and modality (Supplementary Table S9B) of eight parameters. Of the 138 mutants, various data points were available for each parameter (Supplementary Table S9B). As an example, we also show bar plot of the 131 mutants in “actin penetrance” parameter as well as its fitted unimodal beta distribution (Supplementary Figure S15). Eventually, we prepared Supplementary Table S9C describing the eight employed parameters.

## Visualization of the phenotypic correlations for Box-Cox method

To estimate the similarity of phenotypes between the arbitrary pairs of 4,708 non-essential gene deletion mutants, we applied PCA to the Box-Cox transformed data of the 109 WT replicates, the obtained first 88 PCs, covering more than 99% of variance, were used to project Z values of the mutants. Finally, we used these 88 PC scores to calculate the correlation coefficients (*r*) as the phenotypic correlations between mutants. The correlation matrix was employed to construct a similarity network by qgraph function of the qgraph package [13] (Supplementary Figure S10B).

## Morphological similarity of autophagy group

Morphological profiles of 32 member of autophagy group (Group 73; Supplementary Table S5) were compared to one another. To investigate the profile similarity, first, CalMorph values were transformed to Z-values (Wald-test) using the UNIMO pipeline (490 parameters). Then, the obtained Z-values of 109 WT replicates were exposed to PCA. Finally, Z-values of the mutant cells were projected onto PC axes of the WT. Pearson correlation coefficient (*r*) was calculated between each pair using first 94 PC scores (CCR = 99%).

# Supplementary Figures


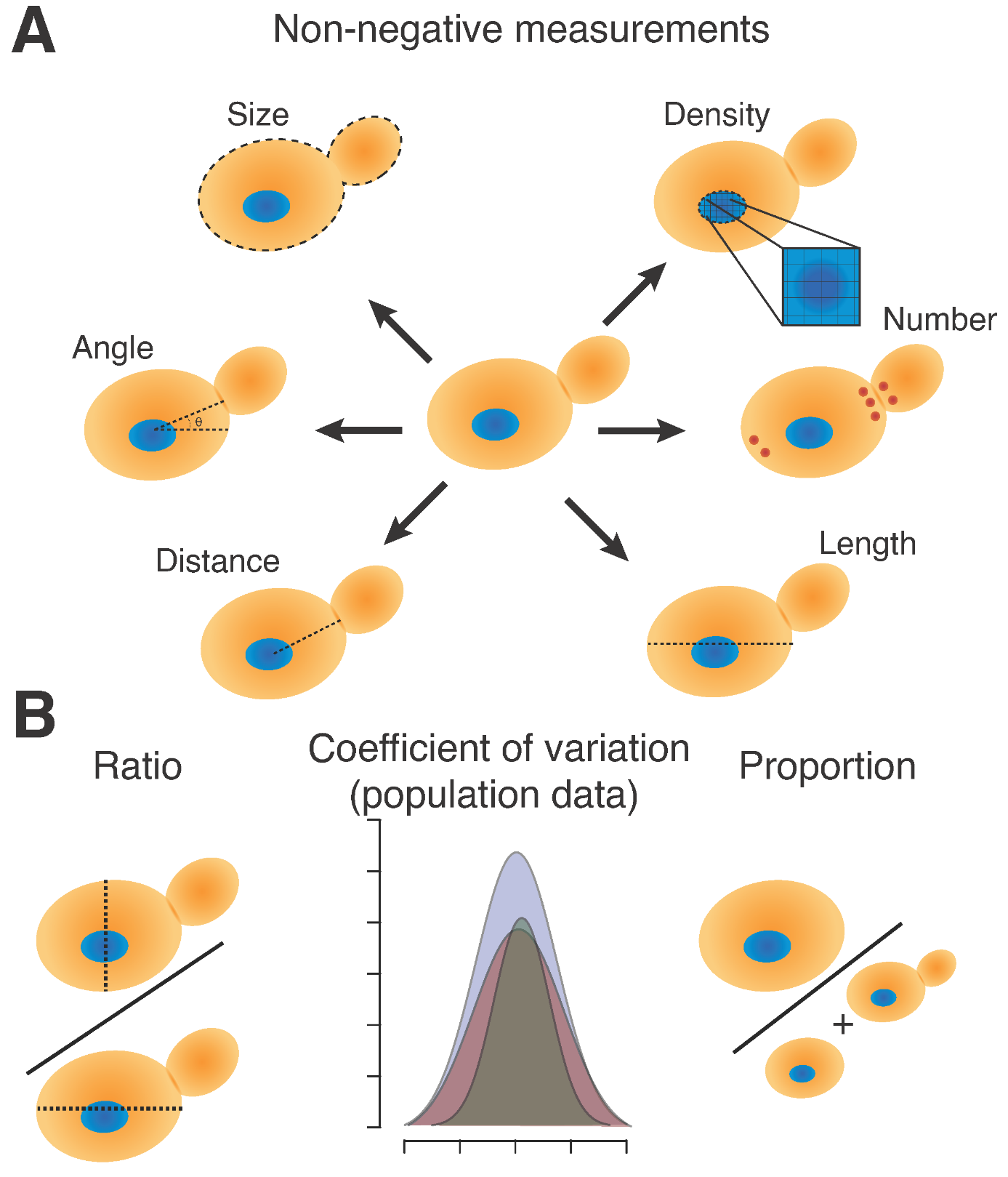


**Supplementary Figure S1. Possible values of cellular features used in QMP.** **(A)** Examples of features defined in yeast cell morphology. Morphometric features (size, shape metrics, and cell architecture), densitometric features (brightness of pixels in each channel of the image within each cellular compartment), and structural/spatial features (angle, distance, and length of objects) all take continuous non-negative values (0 ≤ *y*). The blue oval and red dots illustrate the nucleus and actin patches, respectively. **(B)** Examples of biological measurements associated with yeast cell morphology (i.e., comparison measurements). Continuous bounded values between zero and one (*i.e.*, ratio; 0 ≤ *y* ≤ 1), variations in cell morphology taking continuous positive and negative real values (−∞ < *y* < ∞), and proportions of a specific type of cell taking discrete finite values between zero and one (0 ≤ *y* < 1).


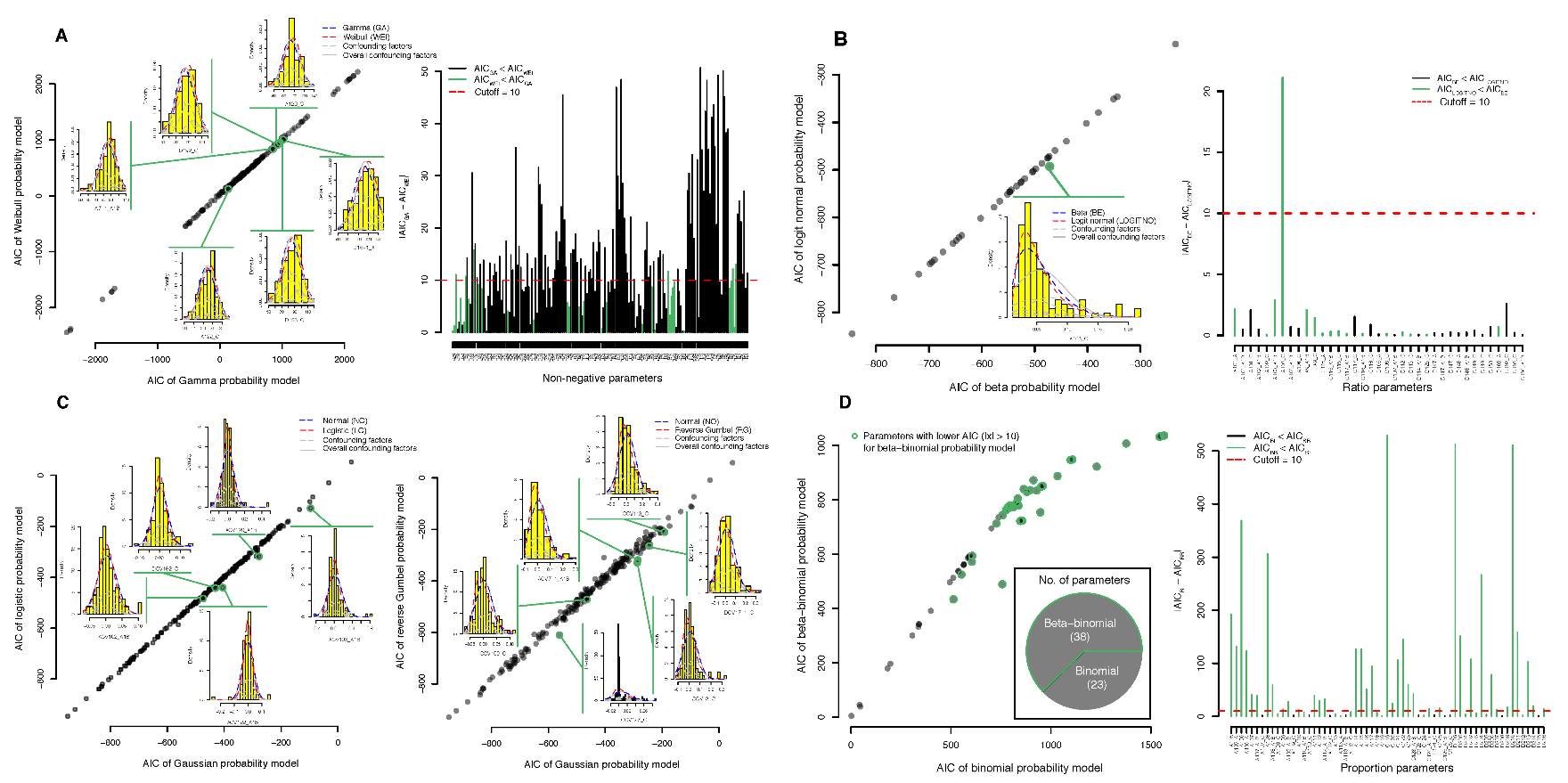


**Supplementary Figure S2. Probability models for CalMorph measures. (A)** The scatter plot shows AIC values of the gamma distribution (as the reference distribution) versus Weibull (the top alternative distribution). Histograms of the fitted distributions and confounding factors when $\left| {AIC}_{Gamma}-{AIC}_{\mathrm{Weibull}} \right|> 10$. Plot of absolute differences of the AICs of the reference (gamma) and alternative (Weibull) distributions (Supplementary Table S1A). **(B)** The scatter plot shows AIC values of the beta distribution (as the reference distribution) versus logit-normal (the top alternative distribution). Histogram of the fitted distributions and confounding factors when $\left| {AIC}_{Beta}-{AIC}_{Logit-normal} \right|> 10$. Plot of absolute differences of the AICs of the reference (beta) and alternative (logit-normal) distributions (Supplementary Table S1B). **(C)** For the 11 non-normal noise parameters, the scatter plots show AIC values of the Gaussian distribution (as the reference distribution) versus logistic and reverse Gumbel, the top alternative distributions (Supplementary Table S1C). **(D)** The scatter plot shows AIC values of the binomial distribution (as the reference distribution) versus beta-binomial (the top alternative distribution). **Inset:** Pie chart of the fitted distributions when $\left| {AIC}_{binomial}-{AIC}_{beta-binomial} \right|> 10$. Plot of absolute differences of the AICs of the reference (binomial) and alternative (beta-binomial) distributions (Supplementary Table S1D).


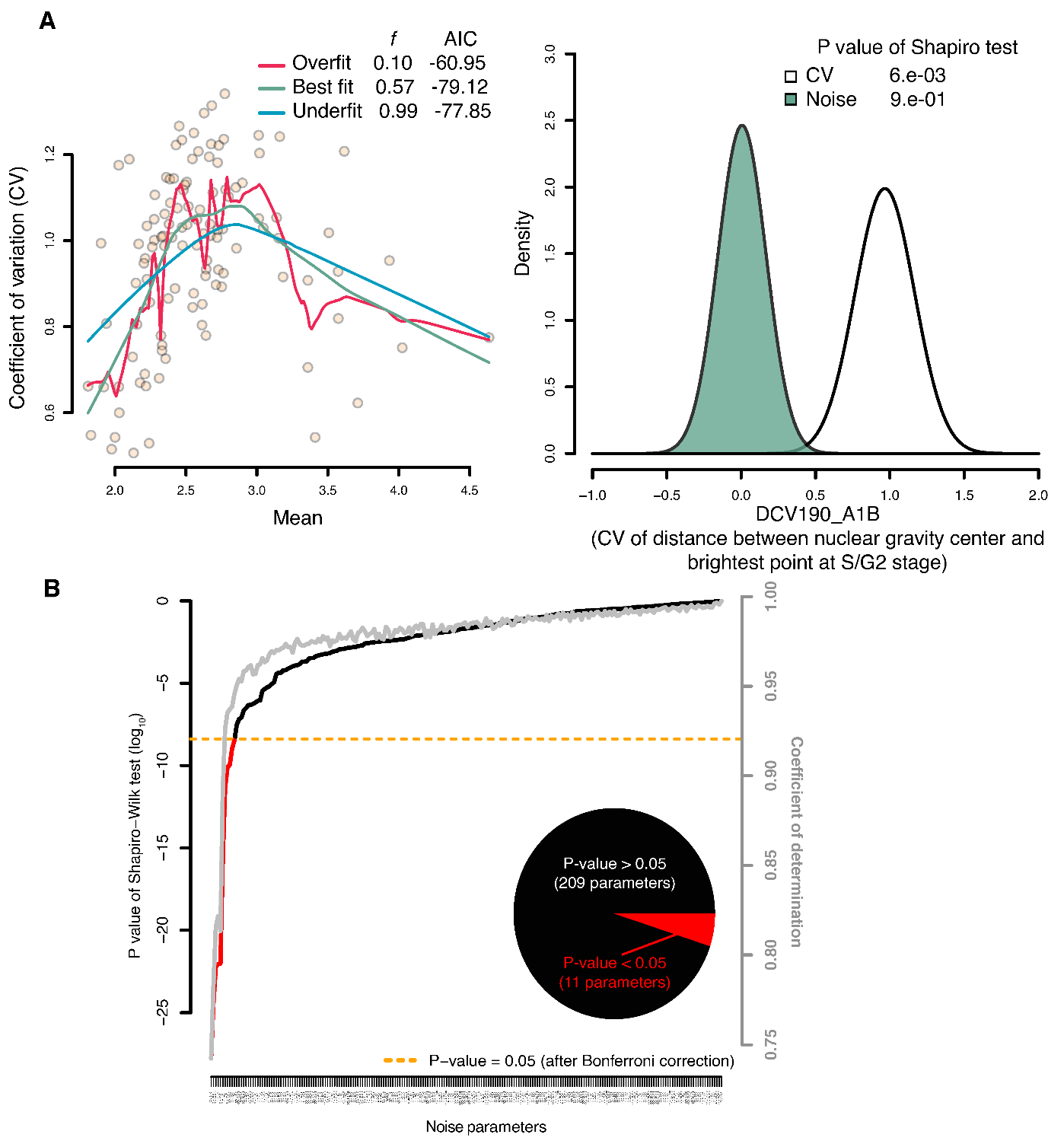


**Supplementary Figure S3. Conversion of the CV to noise values.** **(A)** Left panel: An example of uncoupling the dependency of a CV parameter (DCV190_A1B) and its related means by locally estimated scatterplot smoothing (LOESS) regression. LOESS regression transforms the data around the LOESS fitted curve. *f* is the parameter which controls the degree of smoothing. Right panel: The corresponding distributions of DCV190_A1B before (white) and after transformation to noise values (green) are shown. **(B)** Log_10_ transformed P values of the Shapiro–Wilk normality test revealed that most of the noise parameters (209) are Gaussian distributed (black line). Non-normal parameters are presented in red. The gray line shows *r^2^*, obtained from a QQ normal plot. **Inset:** Number of Gaussian distributed noise parameters given the results of the Shapiro–Wilk normality test after Bonferroni correction.


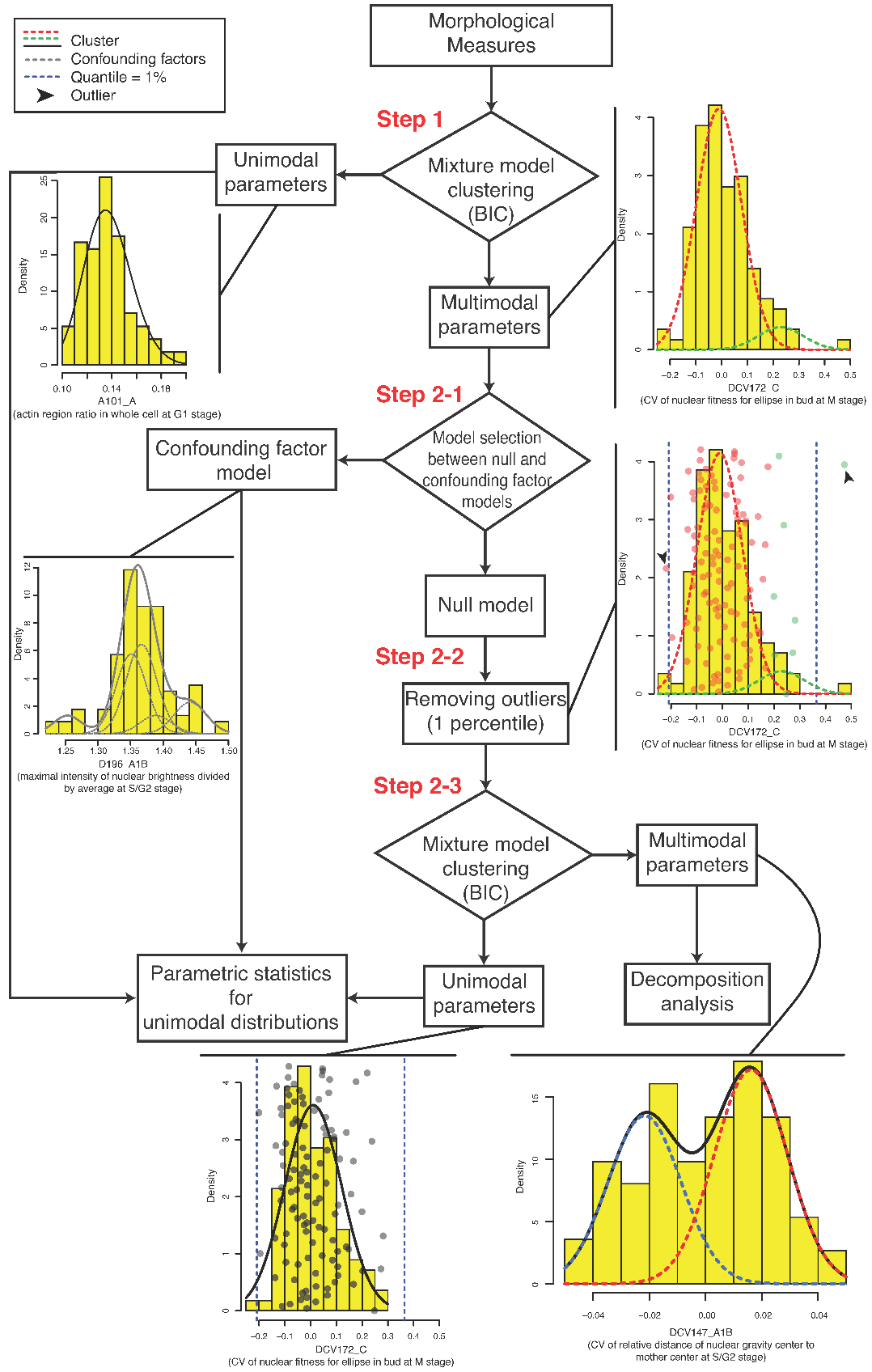


**Supplementary Figure S4. Flowchart of the methodology for checking the modality of the CalMorph parameters.** For the first step, the modality of each CalMorph parameter was checked by mixture model clustering given the predefined probability distributions (Supplementary Table S1). “gamlss.mx” [2] and “mclust” [3] R packages were employed for this aim. Then, confounding factors (Step 2-1; Supplementary Figure S5) and outliers (one-percentile; Step 2-2; Supplementary Figure S6) were considered. To understand the effect of confounding factors and outliers on the data modality. Eventually, each parameter was again exposed to mixture model clustering (Step 2-3). Histograms are shown as examples. The area unit is the number of pixels squared, and the unit for length is the number of pixels; for details, see the CalMorph user manual.


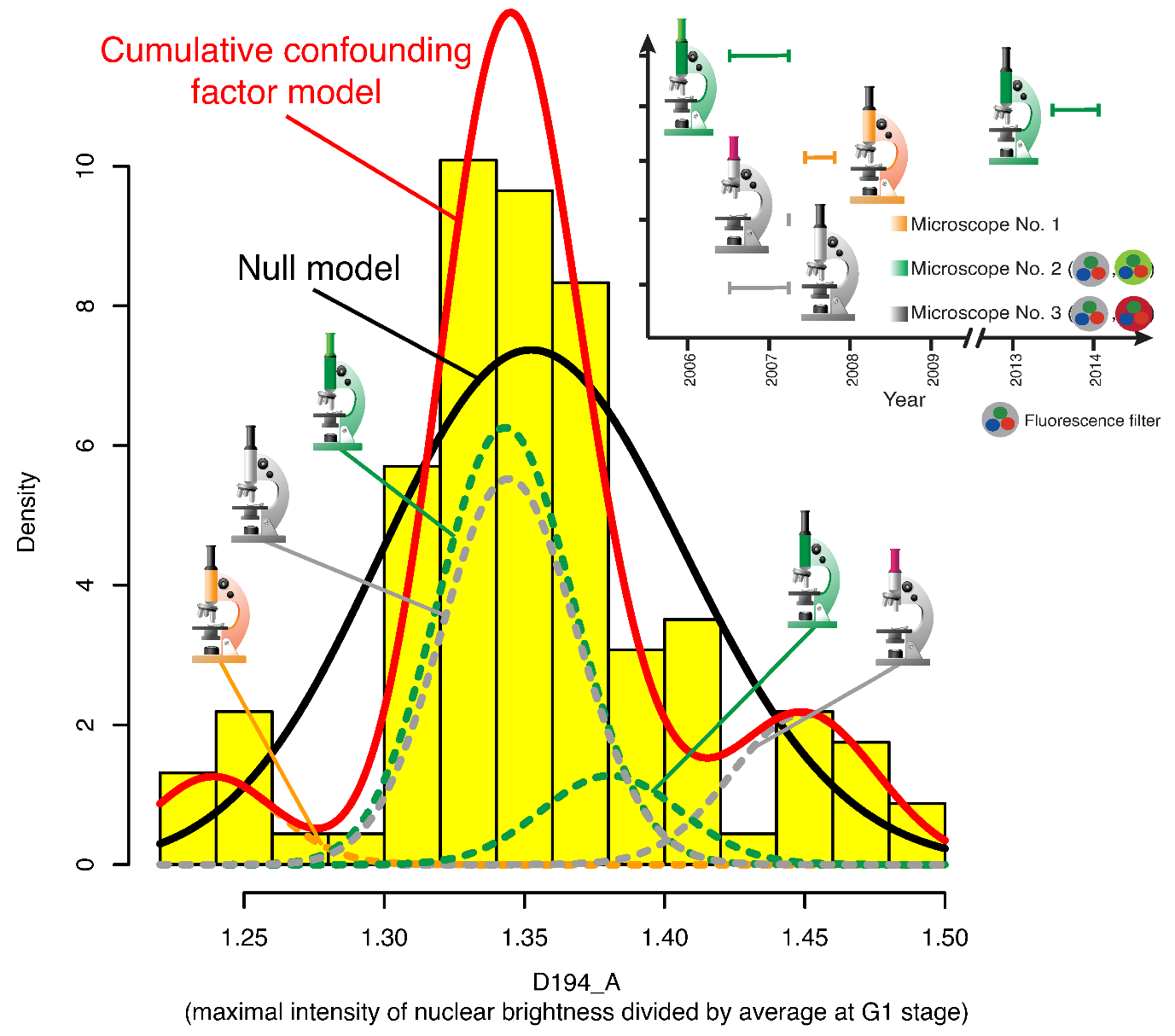


**Supplementary Figure S5. An example of the effect of confounding factors on modality.** Dashed lines represent various experimental conditions (inset). Solid black and red lines show the null and overall distributions based on all confounding factors, respectively. This Figure was designed using resources from [www.freepik.com](http://www.freepik.com).


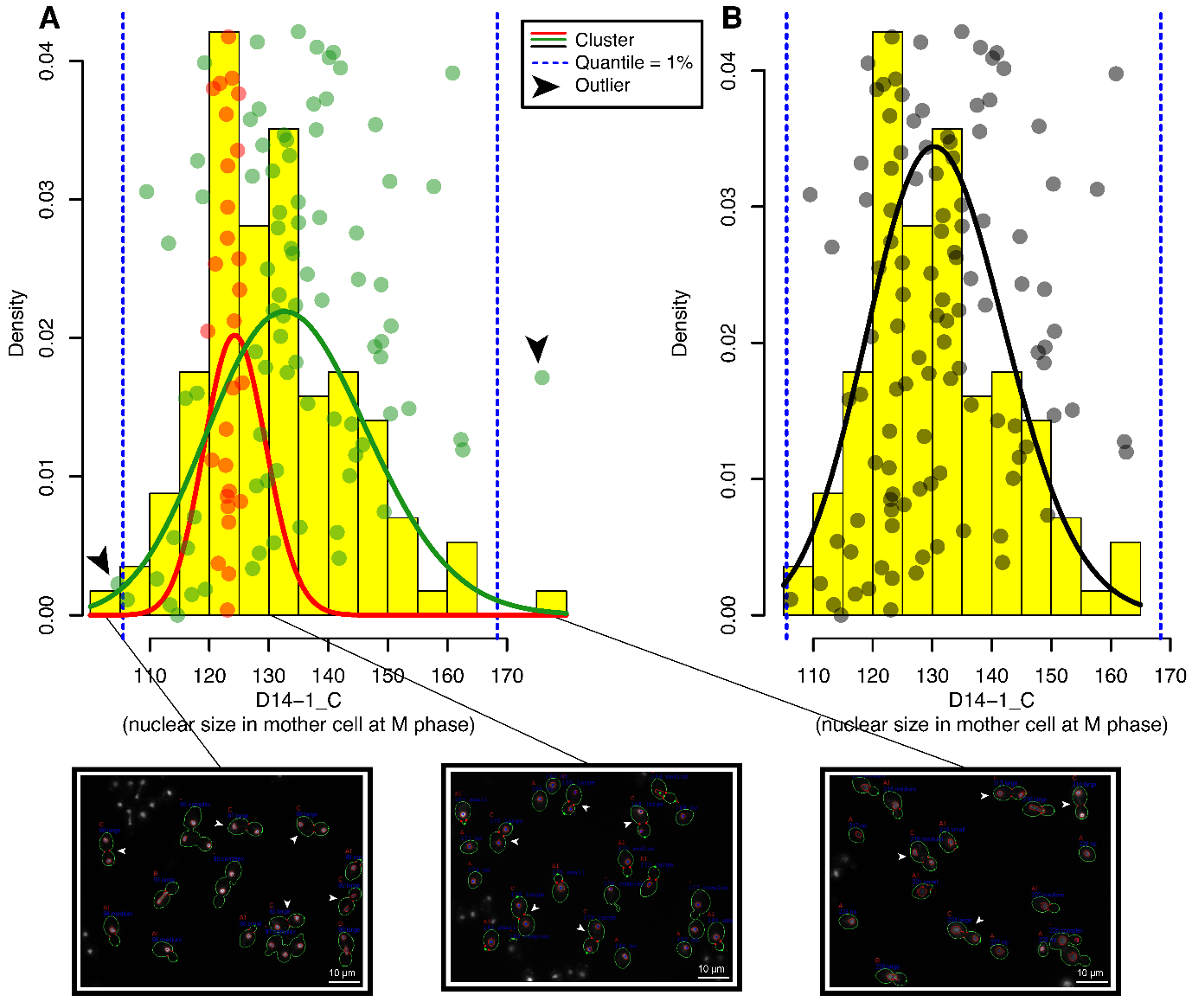


**Supplementary Figure S6. An example of the effect of outliers on modality.** Each circle represents a replicate of the wild-type dataset (n = 114) and is color-coded according to the assigned distributions. The histograms of nuclear size in mother cells at M phase show the distribution of detected clusters before removing the outliers (A) and after removing the outliers (B). After removing the outliers, the data became unimodally distributed (B). Examples of microscopy images (after quantification by CalMorph) are also shown. Actin, cell wall, and nucleus are shown in red, green, and blue, respectively. White arrows point to detected cells which CalMorph used for calculation of D14-1_C. The unit for size is the number of pixels squared; for details, see the CalMorph user manual.


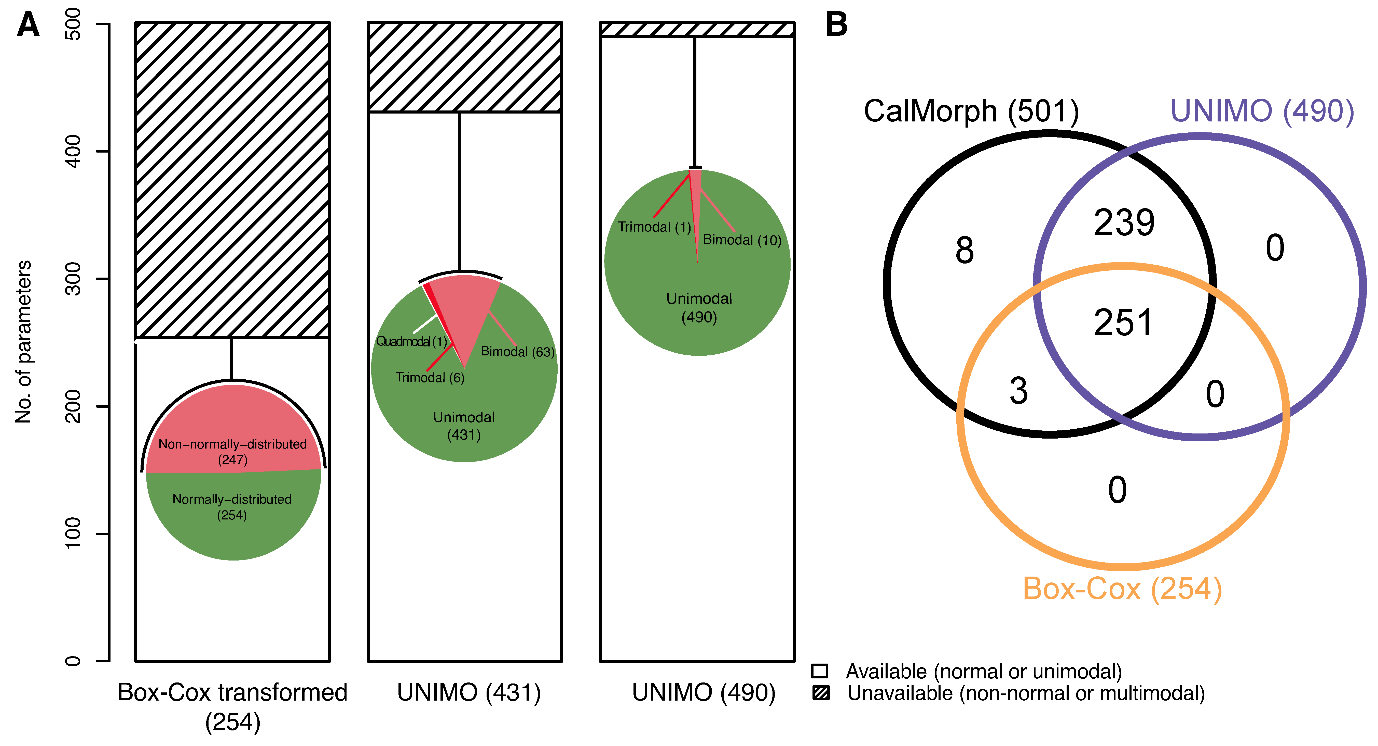


**Supplementary Figure S7. Comparison of this study with our previous results. (A)** Number of available and unavailable parameters during the morphological studies. Box–Cox transformation was employed in the previous QMP [4] to obtain 254 approximately normally distributed parameters. As a result, half of the collected data was not used (Box-Cox). The UNIMO (431) and UNIMO (490) corresponded to the steps in the current study before and after considering confounding factors and deleting outliers, respectively. The green area of the pie charts shows the available parameters; the other parts stand for unavailable parameters. **(B)** Ven diagram of number of available parameters (CalMorph), number of parameters used by UNIMO, and number of parameters used by Box-Cox transformation method. Three shared parameters in Box-Cox method and CalMorph (CCV107_C, D131_C, and DCV182_C) and eight specific parameters in CalMorph (DCV147_A1B, DCV182_A, A123_C, D17-2_C, ACV102_A1B, ACV120_A1B, ACV103_A1B, and CCV13_A) are detected multimodal in this study (see Supplementary Figure S13 and Supplementary Table S3).


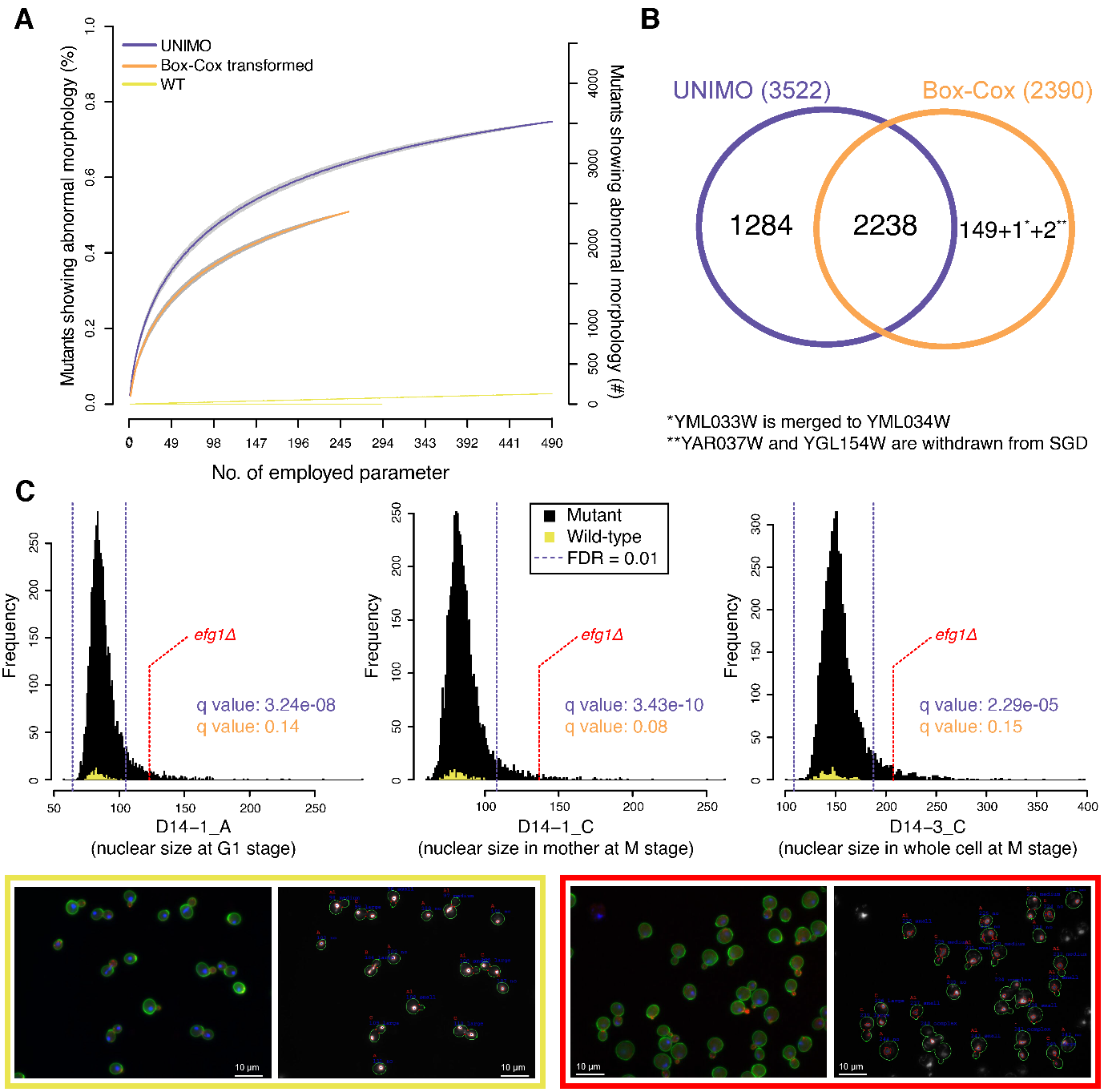


**Supplementary Figure S8. Comparison of results between UNIMO and Box-Cox transformed methods.** **(A)** The percentage (left) and number (right) of mutants exhibiting morphological abnormality in at least one morphological parameter at FDR = 0.01. The Purple and orange curves indicate the results of this study (UNIMO) and our previous report [4] (254 Box–Cox transformed parameters), respectively. The yellow lines indicate false positives (FP) in wild-type replicates. The confidence interval at 95% (gray area) was obtained by 3,000 randomizing iterations. **(B)** Venn diagram of number of significant mutants in both methods. Also see Supplementary Table S4. **(C)** Examples of undetected morphological defects (D14-1_A, D14-1_C, and D14-3_C) of *efg1Δ* (*ygr271c-a*) cells by Box-Cox method. Each histogram represents extracted values by CalMorph for mutants (black) and WT (yellow) cells. Microscopy images (before and after quantification by CalMorph) are also shown for WT cells (yellow box) and *efg1Δ* cells (red box). Actin, cell wall, and nucleus are shown in red, green, and blue, respectively. The unit for size is the number of pixels squared; for details, see the CalMorph user manual.


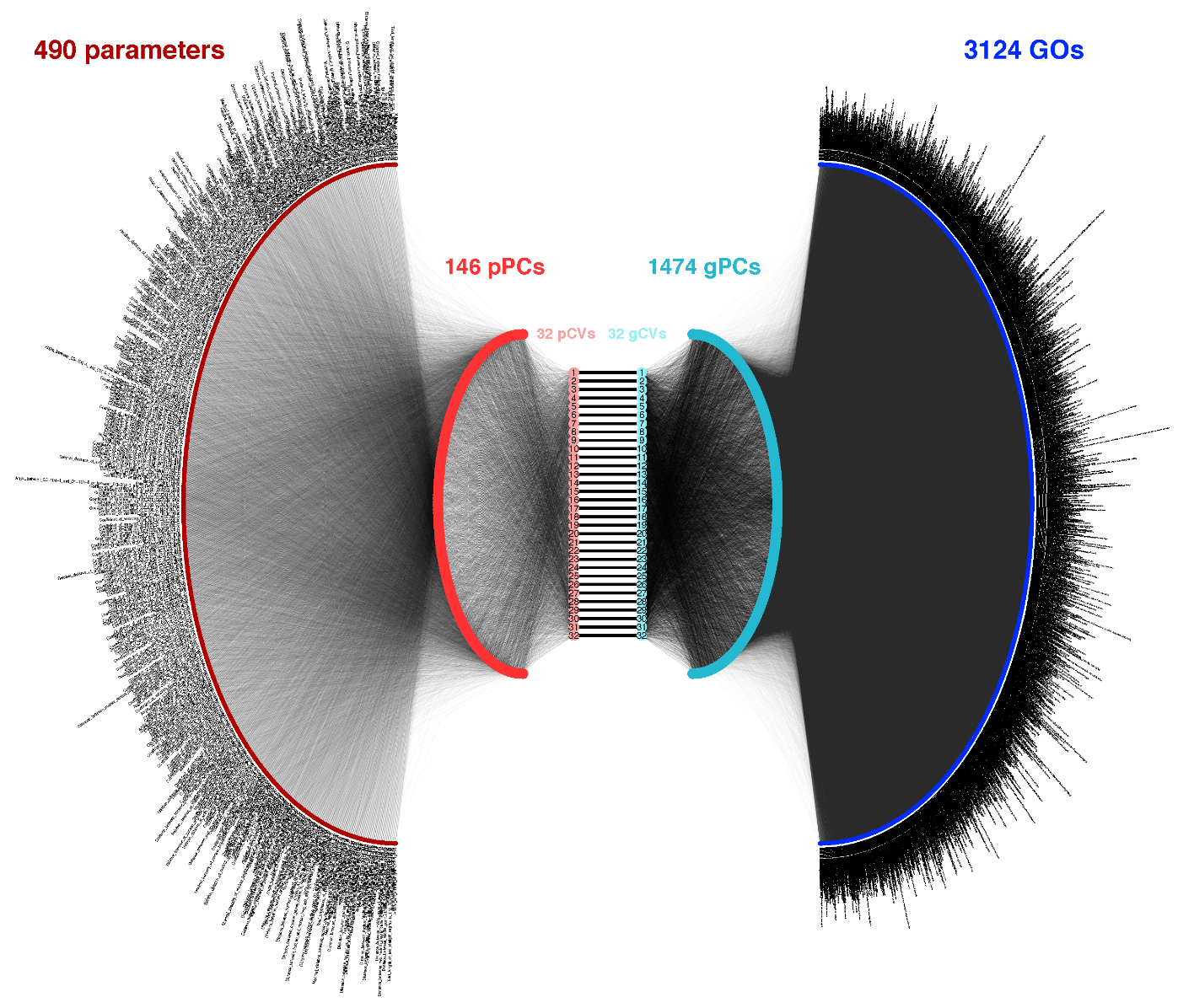


**Supplementary Figure S9. Canonical correlation analysis used for extraction of 32 pairs of canonical variables.** An eye diagram of the CCA procedure. Edges show significant loadings (P < 0.05, t-test) with more than one relationship to other nodes. pPCs: phenotype Principal Components; pCVs: phenotype Canonical Variables; gCVs: GO terms Canonical Variables; gPCs: GO terms Principal Components.


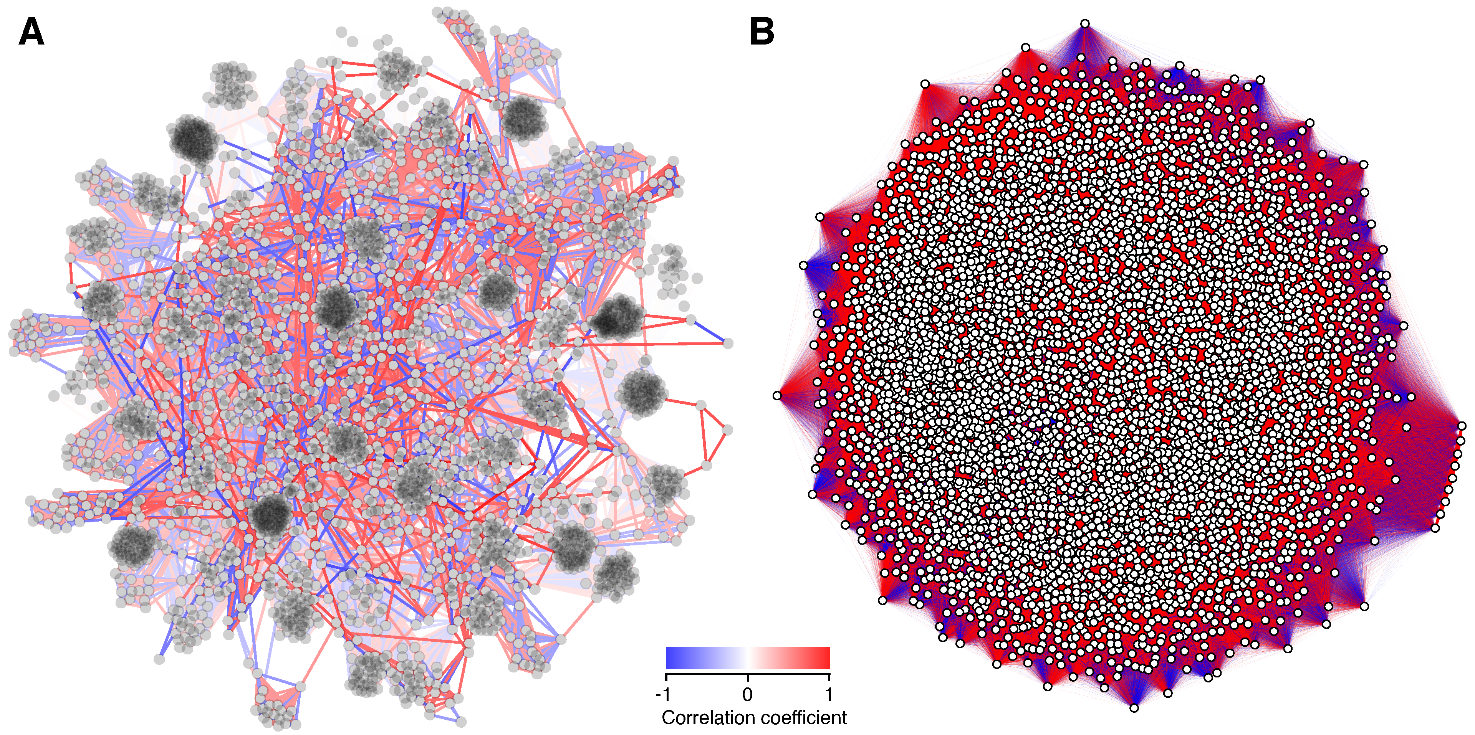


**Supplementary Figure S10. Phenotypic similarity network of non-essential genes.** **(A)** UNIMO method: Graphical representation of yeast non-essential genes (2,945 gray circles) with morphological abnormalities and their functions is shown using the Spring layout (Figure 6A). This network shows similarity of phenotypes between pairs which is calculated using 32 pCV scores and expressed as correlation coefficients (see *Materials and methods*). Of 130 functional groups (P < 0.05; after Bonferroni correction; Supplementary Table S5), 19 core and dense groups are shown. Red and blue lines illustrate positive and negative correlation coefficients (*r*), respectively. **(B)** Box-Cox method: Phenotypic similarity network (4,708 gray circles) according to correlation matrix of 88 PC scores (obtained by projecting Z values of the mutants by first 88 PC scores of the WT replicates; cumulative contribution ratio = 99%) is shown using the Spring layout. Red and blue lines illustrate positive and negative *r*, respectively.


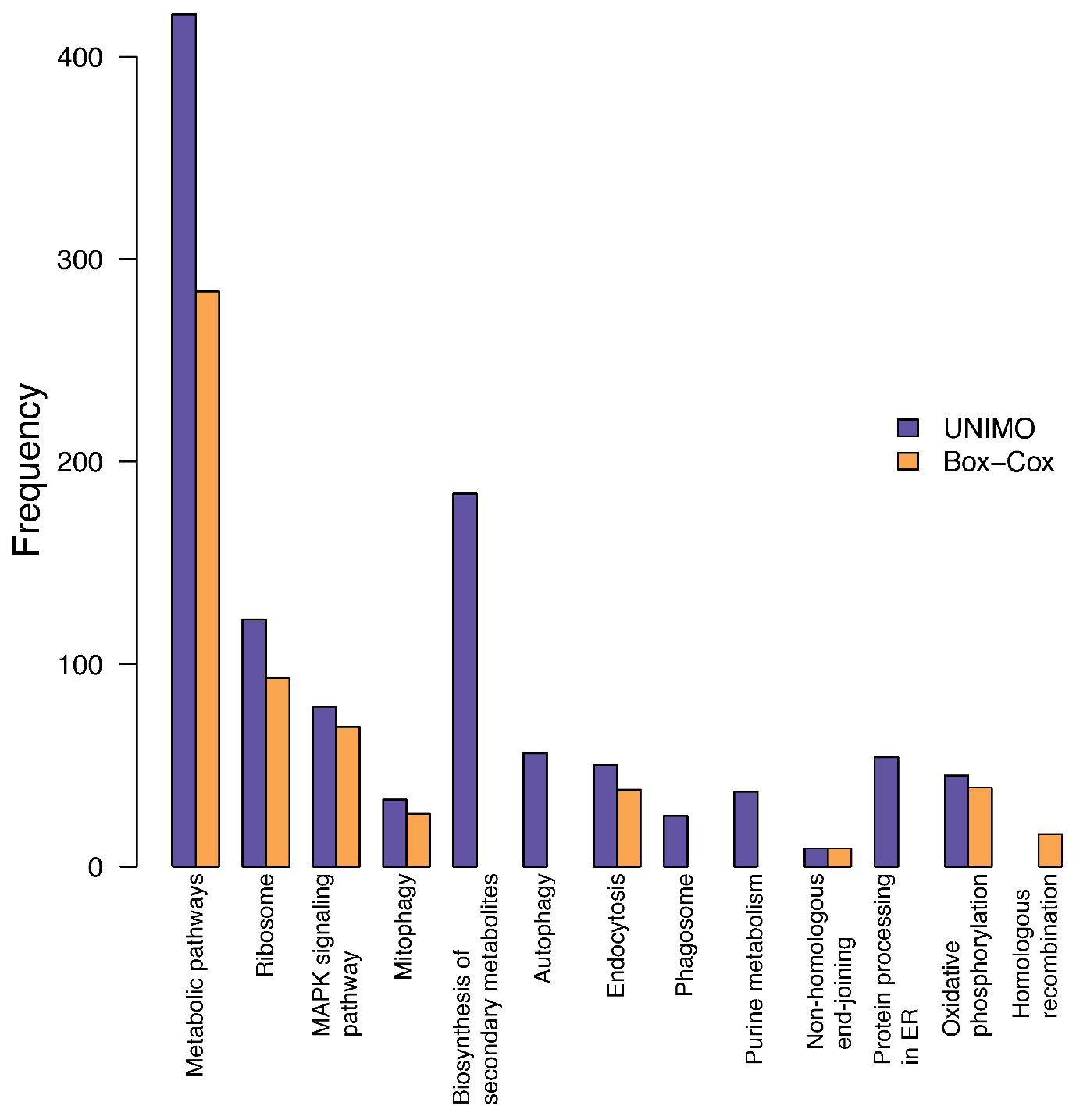


**Supplementary Figure S11. Enrichment of KEGG categories.** Bar plot of KEGG pathway enrichment analysis using significantly abnormal mutants obtained by UNIMO method (3,522) and Box-cox method (2,390). Y-axis shows number of enriched ORFs which are selected at FDR = 0.05. Also see Supplementary Table S6.


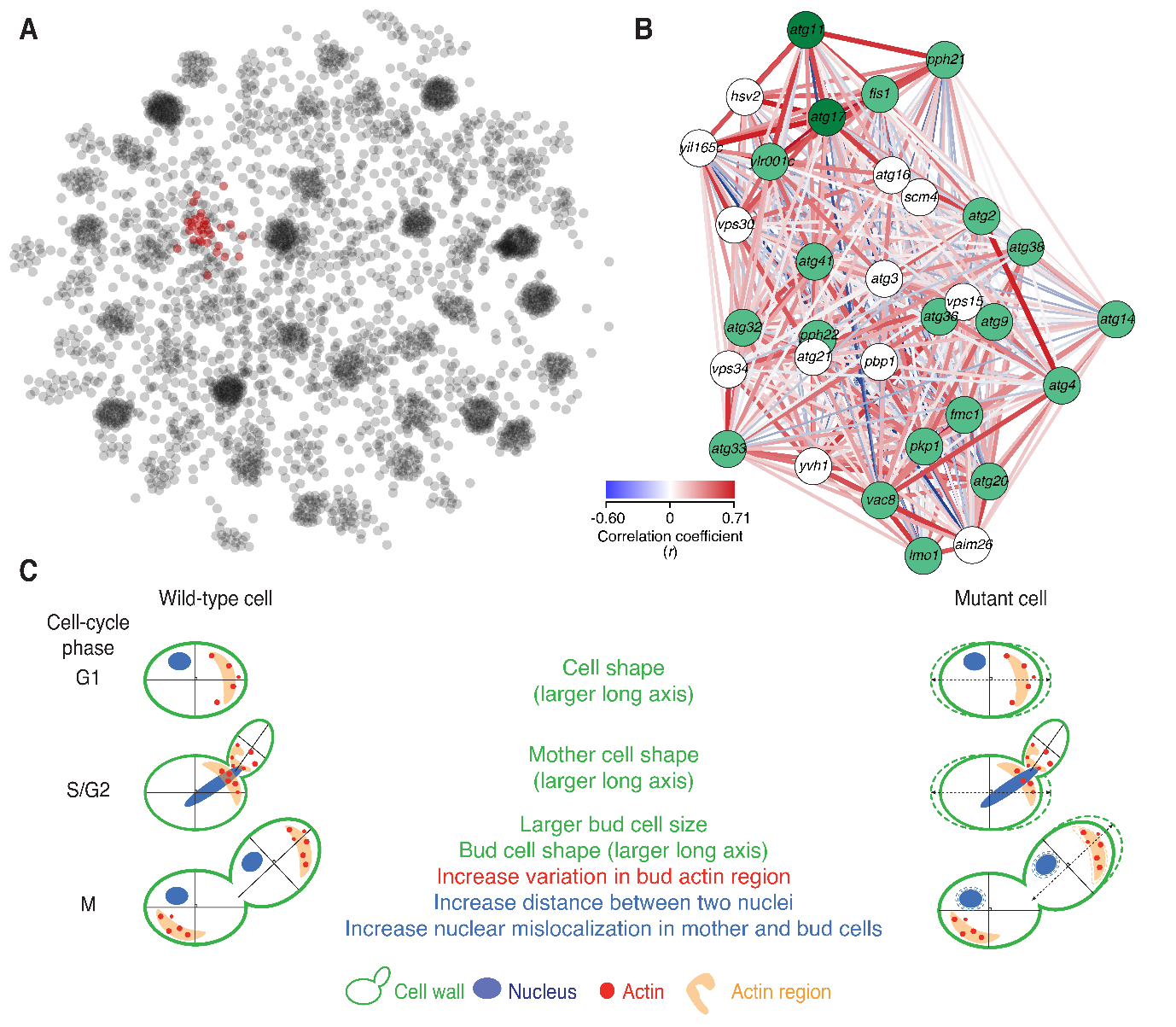


**Supplementary Figure S12. Morphological defects of autophagy mutants. (A)** Graphical representation of yeast non-essential genes with morphological abnormalities and their functions is shown using the Spring layout (Figure 6A and Supplementary Figure S10A). Red circles are 32 genes in functional group 73 (Supplementary Table S5) which are enriched for autophagy (GO ID: GO:0006914). **(B)** A subnetwork corresponding to morphological correlations among 32 autophagy genes (Group 73; Supplementary Table S5) is shown using the Spring layout. Green nodes represent mutants with larger long axis (C115_A; whole cell axis ratio at G1 stage). Red and blue lines show positive and negative correlation coefficients (*r*), respectively. **(C)** Shared effects of *ATG11* or *ATG17* deletion (dark green nodes in “**B**”) on cell morphology are illustrated by schematic budding yeast cells where actin, cell wall, and nucleus are shown in red, green, and blue, respectively.


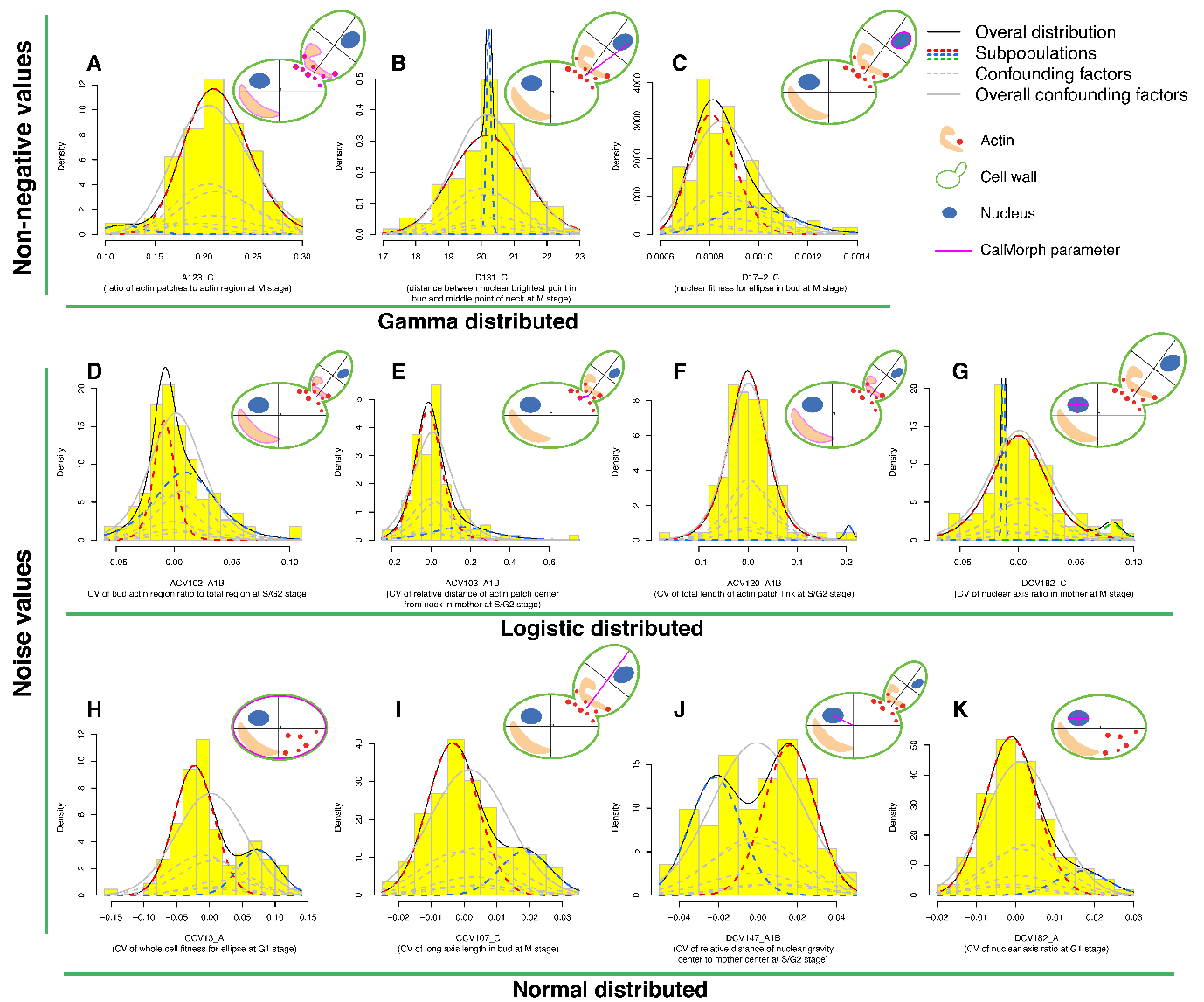


**Supplementary Figure S13. Multimodal CalMorph parameters.** Three non-negative (**A–C**) and eight noise (**D–K**) parameters were identified as multimodal parameters for which the modality could not be explained by known confounding factors or outliers. Dashed red, blue, green, and gray lines show the distributions of subpopulation clusters and confounding factors. Solid gray and black lines show the overall distribution based on all confounding factors and the cumulative distribution of all subpopulations, respectively. The implication of each parameter is illustrated by a schematic budding yeast cell where actin, cell wall, and nucleus are shown in red, green, and blue, respectively. The units for area and length are the number of pixels squared and the number of pixels, respectively. Ratio parameters are unitless; for det ails, see the CalMorph user manual.


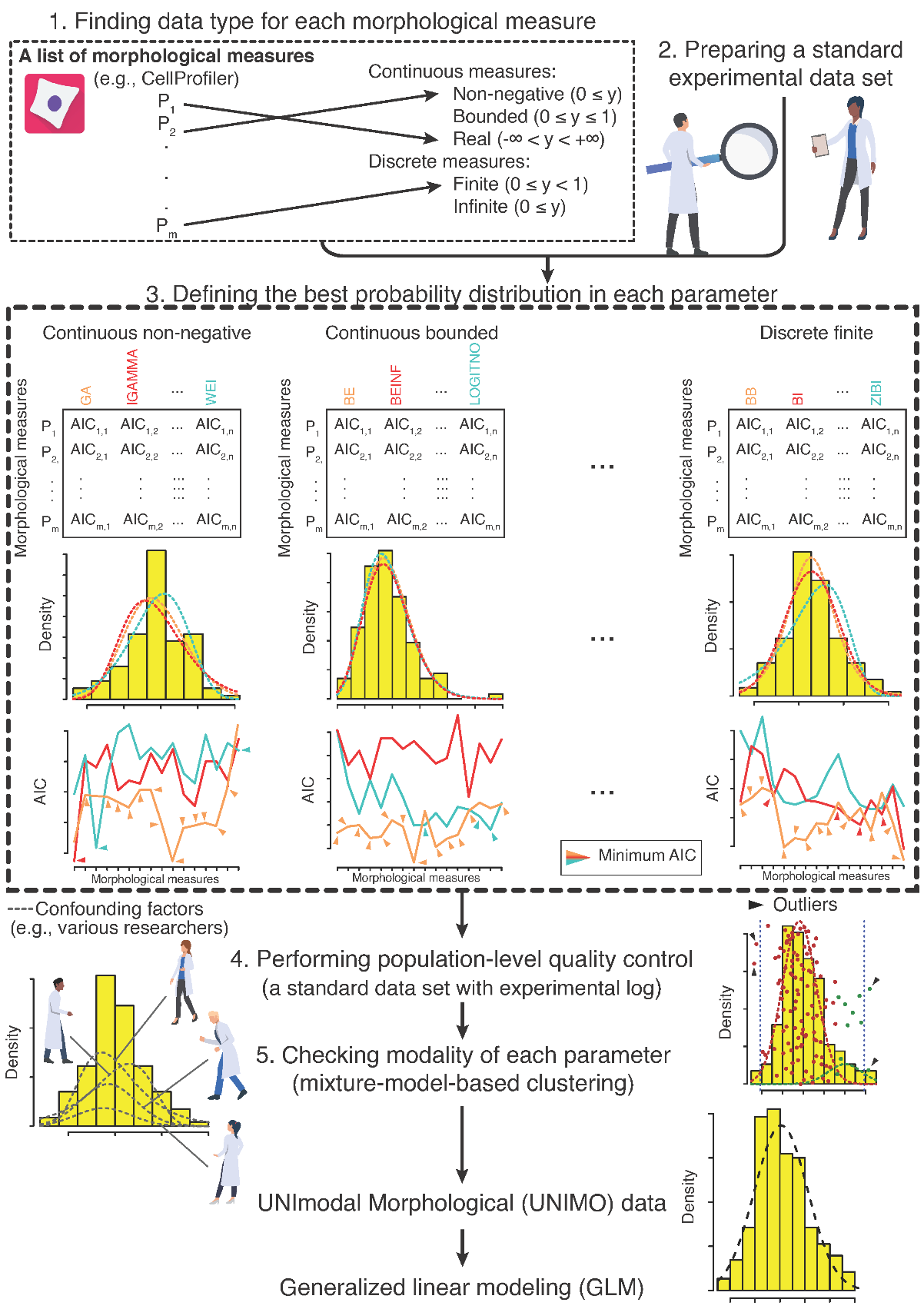


**Supplementary Figure S14. Outlines of generalization of UNIMO.** This figure was designed using resources from [www.freepik.com](http://www.freepik.com).


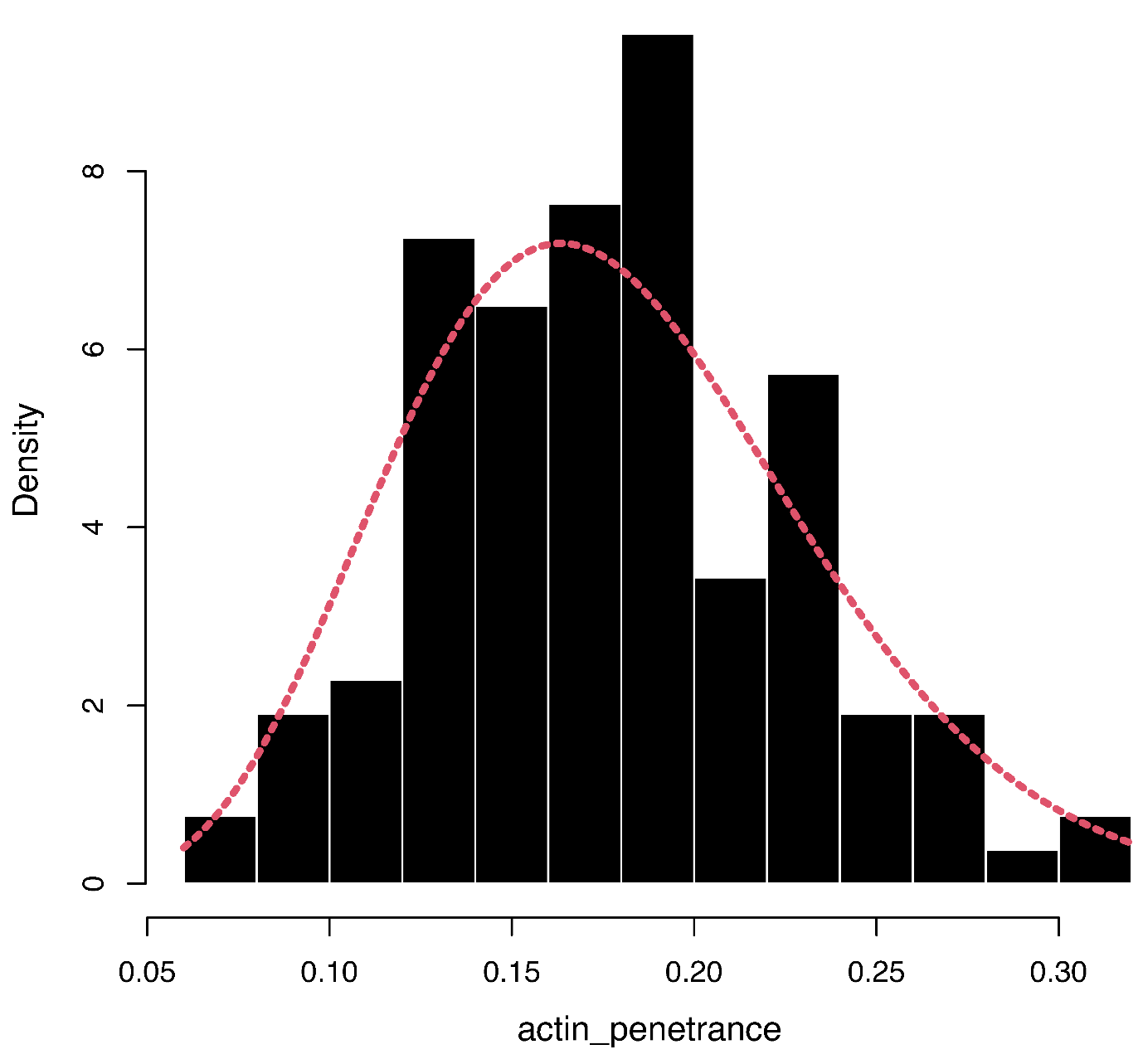


**Supplementary Figure S15. An example of versatility of UNIMO.** Bar plot of actin penetrance in 131 mutants which was obtained from [11]. Dashed line shows unimodal distribution which is obtained from mixture modeling of best pre-defined distribution (beta distribution). Also see Supplementary Table S9A and B.


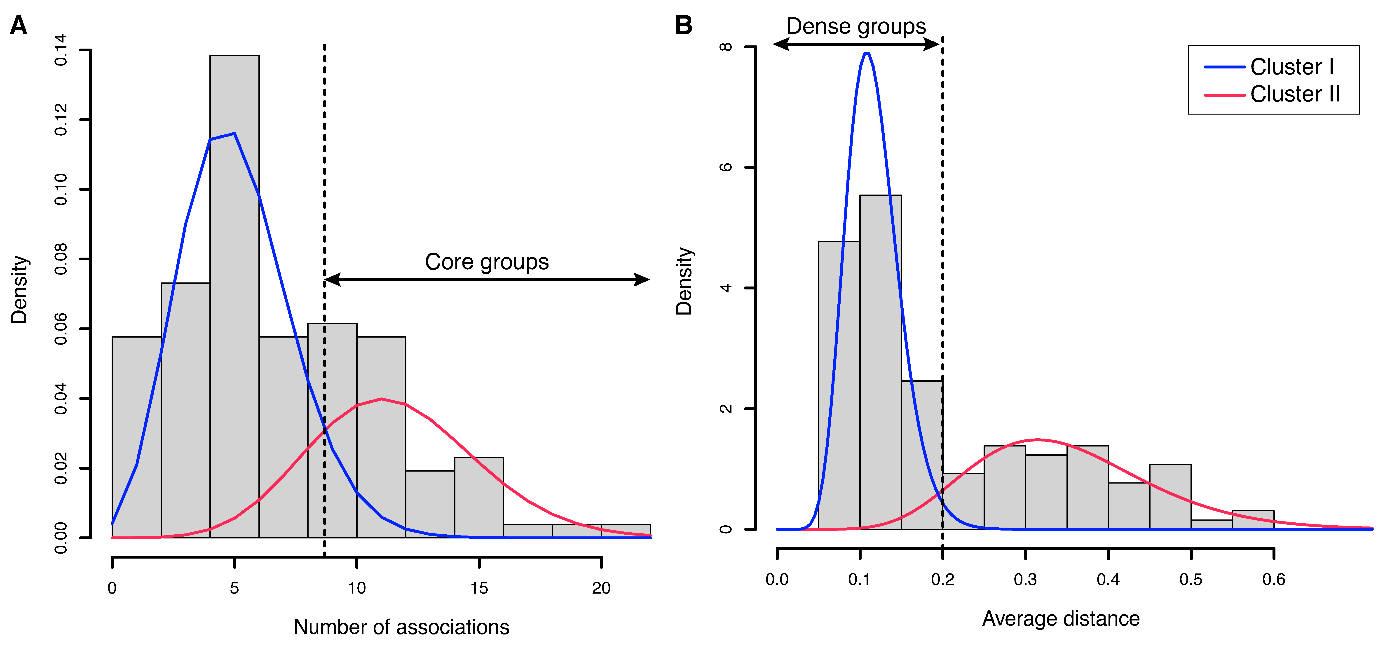


**Supplementary Figure S16. Defining characteristics of the 130 functional groups. (A)** Histogram of the number of the groups with significant correlation(s). Blue and red curves define the underlying populations according to the Poisson mixture-model-based clustering. Core groups are defined as greater than or equal than nine associations. **(B)** Histogram of the average of the distance between arbitrary pairs of genes on the two-dimensional network. Blue and red curves define the underlying populations according to the gamma mixture-model-based clustering. Dense groups are defined as less than 0.2 of the average distance.

# Supplementary Tables

**Supplementary Table S1.** Selection of the probability distribution for each morphological parameter; non-negative (A), ratio (B), noise (C), and proportion (D) measures.

See Table S1.xlsx file.

**Supplementary Table S2.** Population level modality check for each morphological parameter; non-negative (A), ratio (B), noise (C), and proportion (D) measures.

See Table S2.xlsx file.

**Supplementary Table S3.** The best probability distribution and final modality for each morphological parameter; non-negative (A), ratio (B), noise (C), and proportion (D) measures.

See Table S3.xlsx file.

**Supplementary Table S4. List of non-essential mutants.**

See Table S4-9.xlsx file.

**Supplementary Table S5.** List of the representative GO terms enriched in each functional group.

See Table S4-9.xlsx file.

**Supplementary Table S6.** KEGG pathway enrichment (FDR = 0.05).

See Table S4-9.xlsx file.

**Supplementary Table S7.** Data types and probability distributions employed for a typical quantitative morphological phenotyping (QMP) experiment.

See Table S4-9.xlsx file.

**Supplementary Table S8.** List of measurements extracted by CellProfiler.

See Table S4-9.xlsx file.

**Supplementary Table S9.** An example of generalization and versatility of UNIMO using morphological data presented in Mattiazzi Usaj et al. (2020).

See Table S4-9.xlsx file.

# References

1. Stasinopoulos DM, Rigby RA: **Generalized additive models for location scale and shape (GAMLSS) in R**. *J Stat Softw* 2007, **23**(7):1-46.

2. Stasinopoulos M, Rigby B, Stasinopoulos MM, Suggests M: **Package ‘gamlss. mx’**. 2016.

3. Scrucca L, Fop M, Murphy TB, Raftery AE: **mclust 5: Clustering, classification and density estimation using Gaussian finite mixture models**. *R J* 2016, **8**(1):289.

4. Ohya Y, Sese J, Yukawa M, Sano F, Nakatani Y, Saito TL, Saka A, Fukuda T, Ishihara S, Oka S: **High-dimensional and large-scale phenotyping of yeast mutants**. *PNAS* 2005, **102**(52):19015-19020.

5. Levy SF, Siegal ML: **Network hubs buffer environmental variation in *Saccharomyces cerevisiae***. *PLoS Biol* 2008, **6**(11).

6. Yvert G, Ohnuki S, Nogami S, Imanaga Y, Fehrmann S, Schacherer J, Ohya Y: **Single-cell phenomics reveals intra-species variation of phenotypic noise in yeast**. *BMC Syst Biol* 2013, **7**(1):54.

7. Aho K, Derryberry D, Peterson T: **Model selection for ecologists: The worldviews of AIC and BIC**. *Ecology* 2014, **95**(3):631-636.

8. Xu L, Bedrick EJ, Hanson T, Restrepo C: **A comparison of statistical tools for identifying modality in body mass distributions**. *Journal of Data Science* 2014, **12**(1):175-196.

9. Caicedo JC, Cooper S, Heigwer F, Warchal S, Qiu P, Molnar C, Vasilevich AS, Barry JD, Bansal HS, Kraus O: **Data-analysis strategies for image-based cell profiling**. *Nat Methods* 2017, **14**(9):849.

10. Ohnuki S, Ohya Y: **High-dimensional single-cell phenotyping reveals extensive haploinsufficiency**. *PLoS Biol* 2018, **16**(5).

11. Mattiazzi Usaj M, Sahin N, Friesen H, Pons C, Usaj M, Masinas MPD, Shuteriqi E, Shkurin A, Aloy P, Morris Q: **Systematic genetics and single‐cell imaging reveal widespread morphological pleiotropy and cell‐to‐cell variability**. *Molecular systems biology* 2020, **16**(2):e9243.

12. Carpenter AE, Jones TR, Lamprecht MR, Clarke C, Kang IH, Friman O, Guertin DA, Chang JH, Lindquist RA, Moffat J: **CellProfiler: image analysis software for identifying and quantifying cell phenotypes**. *Genome Biol* 2006, **7**(10):R100.

13. Epskamp S, Cramer AO, Waldorp LJ, Schmittmann VD, Borsboom D: **qgraph: Network visualizations of relationships in psychometric data**. *Journal of statistical software* 2012, **48**(1):1-18.
